# Immunothrombosis: A bibliometric analysis from 2003 to 2023

**DOI:** 10.1097/MD.0000000000039566

**Published:** 2024-09-13

**Authors:** Mengyu Hou, Jingxuan Wu, Jiangshuo Li, Meijuan Zhang, Hang Yin, Jingcheng Chen, Zhili Jin, Ruihua Dong

**Affiliations:** a Department of Research Ward, Beijing Friendship Hospital, Capital Medical University, Beijing, China.

**Keywords:** bibliometric, bibliometrix, immunothrombosis, visual analysis, VOSviewer

## Abstract

**Background:**

Immunothrombosis is a physiological process that constitutes an intravascular innate immune response. Abnormal immunothrombosis can lead to thrombotic disorders. With the outbreak of COVID-19, there is increasing attention to the mechanisms of immunothrombosis and its critical role in thrombotic events, and a growing number of relevant research papers are emerging. This article employs bibliometrics to discuss the current status, hotspots, and trends in research of this field.

**Methods:**

Research papers relevant to immunothrombosis published from January 1, 2003, to May 29, 2023, were collected from the Web of Science Core Collection database. VOSviewer and the R package “Bibliometrix” were employed to analyze publication metrics, including the number of publications, authors, countries, institutions, journals, and keywords. The analysis generated visual results, and trends in research topics and hotspots were examined.

**Results:**

A total of 495 target papers were identified, originating from 58 countries and involving 3287 authors from 1011 research institutions. Eighty high-frequency keywords were classified into 5 clusters. The current key research topics in the field of immunothrombosis include platelets, inflammation, neutrophil extracellular traps, Von Willebrand factor, and the complement system. Research hotspots focus on the mechanisms and manifestations of immunothrombosis in COVID-19, as well as the discovery of novel treatment strategies targeting immunothrombosis in cardiovascular and cerebrovascular diseases.

**Conclusion:**

Bibliometric analysis summarizes the main achievements and development trends in research on immunothrombosis, offering readers a comprehensive understanding of the field and guiding future research directions.

## 1. Introduction

Immunothrombosis is a pathological process of intravascular innate immunity formed by the mutual interaction of immune cells and coagulation substances, considered as one of the physiological processes in host defense.^[1]^ Key factors involved in immunothrombosis include platelets,^[2]^ neutrophils,^[3]^ the complement system,^[4]^ and the coagulation factors.^[5]^ Some infectious or noninfectious factors, upon activating the immune system, lead to the interaction of immune cells, especially neutrophils, with activated platelets.^[6]^ Platelets can be activated either directly by the pathogen’s stimulation or through the endothelial system or inflammatory response.^[7]^ The interaction between platelets and leukocytes not only promotes the further recruitment of immune cells but also facilitates platelet adhesion and aggregation, triggering a cascade of coagulation reactions.^[8]^ In addition, the involvement of neutrophil extracellular traps (NETs) and the complement system intensifies the entire process.^[4,9]^ Immunothrombosis arises from the coordinated interaction of the immune system, complement system, and coagulation system.

Under normal circumstances, intravascular immunothrombosis is beneficial for capturing and clearing pathogens invading the bloodstream. However, uncontrolled immunothrombosis leads to pathological thrombus formation.^[10]^ Dysregulated inflammatory states such as sepsis,^[11]^ systemic lupus erythematosus (SLE),^[12]^ acute respiratory distress syndrome,^[13]^ stroke,^[14]^ venous thromboembolism (VET),^[15]^ and coronary artery disease,^[16]^ leads to progressive thrombus formation. In excessive inflammation, inflammasomes promote coagulation triggered by tissue factor (TF), while neutrophils-platelets interaction fosters NETs and thromboxane A2 formation, resulting in thrombosis.^[15]^

The outbreak of COVID-19 has shifted researchers’ focus towards immunothrombosis, as it has been demonstrated to play a significant role in the disease progression and adverse outcomes in COVID-19 patients.^[17]^ In recent years, with an increasingly in-depth study of immunothrombosis in COVID-19, the roles of NETs, inflammatory factors, the complement system, endothelial system, and TF in immunothrombosis have been more clearly elucidated.^[4,18]^

Bibliometrics is a discipline that involves reviewing literature to predict the development of scientific research in a particular field.^[19]^ Bibliometrics can efficiently and accurately summarize the development and trends in research content within a specific field, thus providing guidance for subsequent work.^[20]^ This article utilized data from articles related to immunothrombosis recorded in the Web of Science Core Collection (WOSCC) database from 2003 to 2023. The analysis involved 495 target documents using VOSviewer and the Bibliometrix R package. The development process, current status, frontier discoveries, and future trends in the field of immunothrombosis were analyzed from the perspectives of basic data, the information of co-occurrence relationships, and the cluster analysis. The aim of this study is to provide reference points for subsequent research in this field.

## 2. Materials and methods

### 2.1. Data sources and filtration

This study collected literature from the WOSCC database for analysis. The Web of Science is the most commonly used database for bibliometric analysis, and its recorded data is considered the most comprehensive and reliable.^[21]^ The literature retrieval strategy for this study was “(((((TS = (Immunothrombosis)) OR TS = (Thromboinflammation)) OR TS = (Thromboinflammatory)) OR TS = (immunothrombus)) OR TS = (Immunothrombotic)) OR TS = (Thromboplasminflammation),” with the index date set from January 1, 2003, to May 27, 2023. The document types selected were “Article,” “Review Article,” or “Early Access.” After cleaning the initially retrieved literature data, articles with insufficient relevance to the research content in the field of immunothrombosis and duplicate publications were excluded, resulting in a total of 495 target papers.

### 2.2. Bibliometric analysis

Apply VOSviewer to gather basic information on authors, countries, institutions, sources, citations, keywords, and references from the target papers.^[22]^ Utilize the Bibliometrix R package to compile information on the annual publication volume of target papers, distribution of authors’ nationalities, core journals, and the evolution trend of keywords, along with thematic information.^[23]^

## 3. Results

A total of 686 publications in the past 20 years were preliminarily included, then 495 publications (284 articles and 211 reviews) were selected after screening. The 495 publications were published in 223 journals with 3287 authors from 1011 research institutions in 58 countries, citing a total of 27,940 references.

### 3.1. Publications

**Figure 1.**
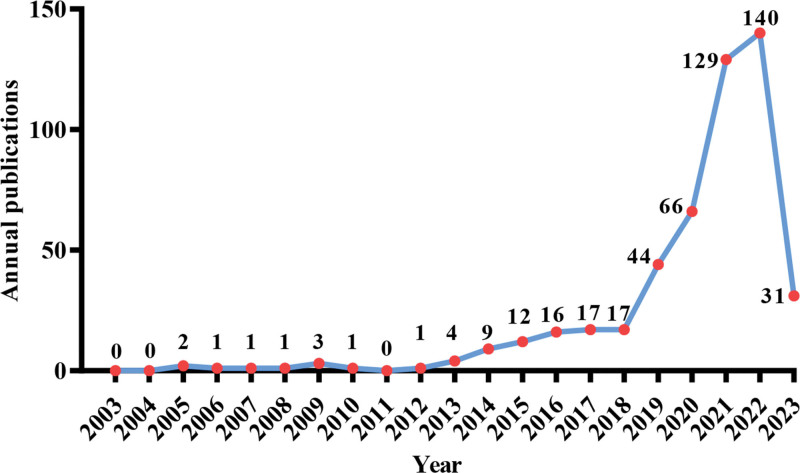
Number of annual publications in the field of immunothrombosis.

Over the past 2 decades, there has been an overall upward trend in the publication volume of papers within the field of immunothrombosis. Papers related to immunothrombosis were first published in 2005, but before 2015, the annual publication volume in this field was consistently below 10 papers per year. After 2015, there has been a steady increase in the annual publication volume, with a rapid surge observed from 2019 onwards, reaching the highest value in 2022. Figure 1 illustrates the temporal distribution of paper publication volume in the field of immunothrombosis.

### 3.2. Authors

According to the Price Law,^[24]^ authors with no <3 papers were defined as the core authors in the field of immunothrombosis, resulting in a total of 141 core authors. Table 1 presents information on the top 5 most productive authors in terms of publication volume in this field.

**Table 1:** Publication information for high-productivity core authors.

Rank	Author	Documents	Citations	Average citation	H-index
1	Bo Nilsson	12	644	53.7	12
2	Kristina N Ekdahl	10	458	45.8	10
3	Steffen Massberg	9	2107	234.1	9
4	Jason S Knight	9	245	27.2	7
5	Tom Eirik Mollnes	9	49	5.4	3

**Figure 2.**
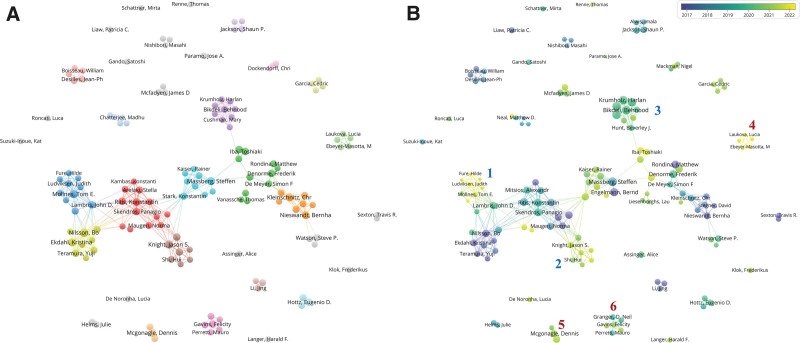
Authors’ cooperation analysis. (A) Nodes with the same color belong to the same cluster, the size of nodes represents the publication volume, and the thickness of connecting lines indicates the number of collaborations. (B) Node size represents the total citation count for each author, node color indicates the average time between publications, with colors closer to yellow indicating more recent publications. The thickness of lines between nodes represents the number of collaborations between authors.

To further clarify the collaboration among core authors, VOSviewer was used to generate a co-authorship knowledge map, illustrating their collaboration relationships as shown in Figure 2. The 141 core authors were categorized into 34 clusters, with 25 being independent clusters that did not collaborate with each other. The remaining 9 clusters collaborated, forming one large research group. Analysis of Figure 2A reveals that within this large research group, there are collaborative relationships among clusters represented by core authors such as Bo Nilsson, John D. Lambris, Konstantinos Ritis, Jason S. Knight, Steffen Massberg, Behnood Bikdeli, Bernhard Nieswandt, and Frederik Denorme.

Analysis of Figure 2B indicates that within the largest collaborative group, there are significant differences in the average publication time among various research teams. Clusters represented by Bo Nilsson and Bernhard Nieswandt had an average publication time before 2018, indicating early involvement in this field. Steffen Massberg individual average publication time is in 2018, but among the collaborators, the average publication time is later than 2020. This suggests that Steffen Massberg, as one of the early pioneers in the field, has facilitated the formation of newer research groups. Groups led by Tom Eirik Mollnes, Jason S. Knight, and Behnood Bikdeli have an average publication time after 2020, indicating later engagement in the field.

The research focus of Tom Eirik Mollnes’ team (group 1) mainly involves the role of the complement system in promoting thrombosis and inflammation.^[25,26]^ Behnood Bikdeli group (group 3) focuses on the clinicopathologic features of thrombosis in COVID-19 patients,^[27]^ treatment strategies,^[28]^ and the clinical application of anticoagulants in COVID-19 patients.^[29]^ In addition, Behnood Bikdeli has an average citation per paper of 426.0, garnering widespread attention.

Among independent research groups, the group represented by Marie Ebeyer-Masotta (group 4) has the most recent average publication year, which is April 2022. Among the emerging research groups formed after 2020, the group represented by Jason S. Knight (group 2) has the highest total publication volume. Their research primarily focuses on the mechanisms and treatment strategies of immunothrombosis in antiphospholipid syndrome (APS).^[30–32]^ Dennis McGonagle (group 5) has an average publication year of 2021 and has contributed 8 papers to this field, with a total citation of 644. His research focuses on the immunological mechanisms, disease characterization, and treatment targets of immunothrombosis in COVID-19.^[33–35]^ Felicity N. E. Gavins (group 6), as a corresponding author, has published 6 articles, with research mainly related to the discovery of treatment targets for immunothrombosis.^[36–38]^

### 3.3. Countries and institutions

**Figure 3.**
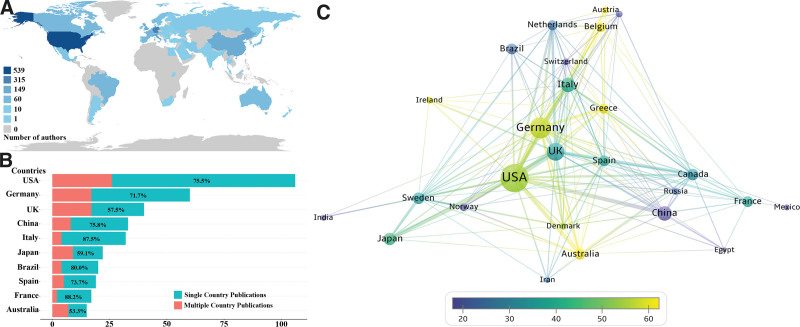
Analysis of the cooperation between countries. (A) Global distribution of the occurrence frequency of authors’ institutions. (B) Multiple country publications and Single country publications number of corresponding authors in the top 10 most productive countries. (C) Network visualization map of countries cooperation. The size of the node represents the number of publications, the thickness of the link represents the amount of cooperation between 2 countries, and the color of the node represents average citation per paper of the country.

This study conducted a statistical analysis of publication information for 58 countries involved in the field of immunothrombosis, visualizing and analyzing the collaboration among countries with more than 5 papers. Table 2 presents the top 5 high-productivity countries in terms of publication volume. The cooperation between countries with more than 5 papers was showed in Figure 3. Figure 3A illustrates the global distribution of the frequency of author affiliation appearances, with research institutions located in the United States appearing most frequently, followed by Germany and Italy. This suggests that researchers affiliated with institutions in these countries are more active in the field of immunothrombosis. Figure 3B shows the country-wise distribution based on the corresponding author’s location. Among the top 10 countries in terms of publication volume, the number of single country publications (SCP) and multiple country publications and the SCP ratio (SCP/total articles) are presented. Researchers from Australia (SCP ratio: 53.3%) and the UK (SCP ratio: 57.5%) tend to initiate international collaborations involving multiple institutions, while researchers from Italy (SCP ratio: 87.5%) and France (SCP ratio: 88.2%) prefer collaboration with researchers from their own country.

**Table 2:** High-productivity country information.

Rank	Country	Publications	Citations	Average citation	Number of collaborations
1	USA	156	8298	53.2	24
2	Germany	88	4954	56.3	23
3	UK	63	2151	34.1	23
4	Italy	43	1671	38.9	20
5	China	41	731	17.8	13

To better analyze the collaboration between countries, Figure 3C presents a co-occurrence map of countries with a total publication volume of at least 5 papers. The United States, the United Kingdom, Germany, and Italy engage in frequent collaborations with multiple countries, with U.S. researchers being the most active in participating in international collaborative research. Additionally, it can be seen that the top 3 countries in terms of average citation per paper, Greece, Australia, and Belgium, have 19, 16, and 13 collaborating countries, respectively (an additional file shows this in more detail, see Table S1, Supplemental Digital Content, http://links.lww.com/MD/N522). This information suggests that participating in multi-country collaborations is beneficial for enhancing research quality.

**Figure 4.**
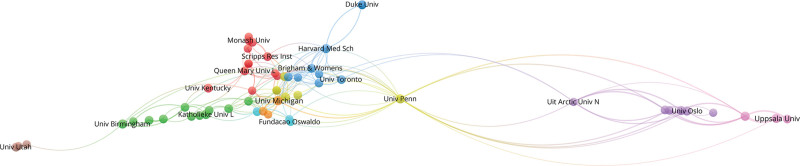
Network visualization map of institutions cooperation.

Analyzing the affiliations of authors can provide further insights into the background of research teams in the field of immunothrombosis. Table 3 compiles information on the top 11 institutions by publication volume. To better analyze the collaboration between research institutions, a co-occurrence knowledge graph was generated for institutions with a publication volume of at least 5 papers (Fig. 4). Among the 59 eligible institutions, 56 institutions have formed collaborative relationships. All of the top 11 most productive institutions are all prestigious traditional institutions in Europe and the United States.

**Table 3:** Information on research institution publication volume.

Rank	Organization	Publications	Citations	Average citation
1	University of Michigan	16	902	56.4
2	University of Uppsala	16	600	37.5
3	Linnaeus University	14	481	34.4
4	University of Pennsylvania	13	1132	87.1
5	University of Birmingham	12	506	42.2
6	Harvard Medical School	11	1963	178.4
7	University of Oslo	11	59	5.4
8	Democritus University of Thrace	10	1087	108.7
9	KU Leuven	10	664	66.4
10	University of Leeds	10	648	64.8
11	Norwegian University of Science and Technology	10	49	4.9

### 3.4. Sources

**Figure 5.**
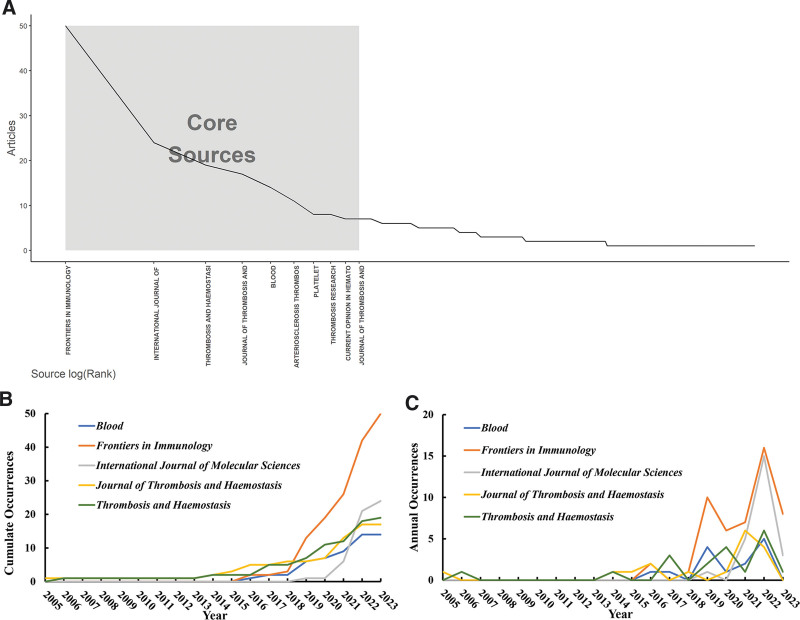
Core journals in the field of immunothrombosis. (A) Core journals identified by the Bradford law. (B) The cumulative publication volume of the top 5 core journals from 2005 to 2023. (C) The annual average publication volume of the top 5 core journals from 2005 to 2023.

This study analyzed 495 target papers published in 223 journals, with 36 journals having a publication volume of at least 3 papers, totaling 275 published articles, accounting for 55.6% of the total papers in this field. Table 4 displays the top 5 journals by publication volume, all of which belong to the Web of Science Journal Impact Factor Quartile 1 journals, with a focus on hematology. Figure 5 presents the basic information of core journals in the field of immunothrombosis. Using Bradford Law, bibliometrix identified the core journals in this field, as shown in Figure 5A.

**Table 4:** Core journal information.

Rank	Journal	Publications	Citations	Average citation	Impact factor	H-index	Journal impact factor quartile/rank	Proportion of open-access publications
1	*Frontiers in Immunology*	50	1303	26.06	8.787	20	Q1(35/162)	99.52%
2	*International Journal of Molecular Sciences*	24	176	7.33	6.208	7	Q1(69/297)	96.25%
3	*Thrombosis and Haemostasis*	19	483	25.42	6.830	9	Q1(18/78)	14.26%
4	*Journal of Thrombosis and Haemostasis*	17	942	55.41	16.041	12	Q1(5/78)	24.83%
5	*Blood*	14	1375	98.21	25.669	9	Q1(2/78)	2.24%

Figure 5B illustrates the cumulative publication volume of the top 5 core journals from 2005 to 2023, with *Frontiers in Immunology* having the highest overall publication volume, while the other 4 core journals show no significant differences in publication volume. Figure 5C presents the annual average publication volume of core journals, highlighting that the *Journal of Thrombosis and Haemostasis* was the earliest to publish articles in this field (since 2005). Since 2018, *Frontiers in Immunology* has consistently had the highest annual publication volume. Notably, the *International Journal of Molecular Sciences* contributed mostly in 2022, making it a relatively new journal in the core group.

### 3.5. Keywords

**Figure 6.**
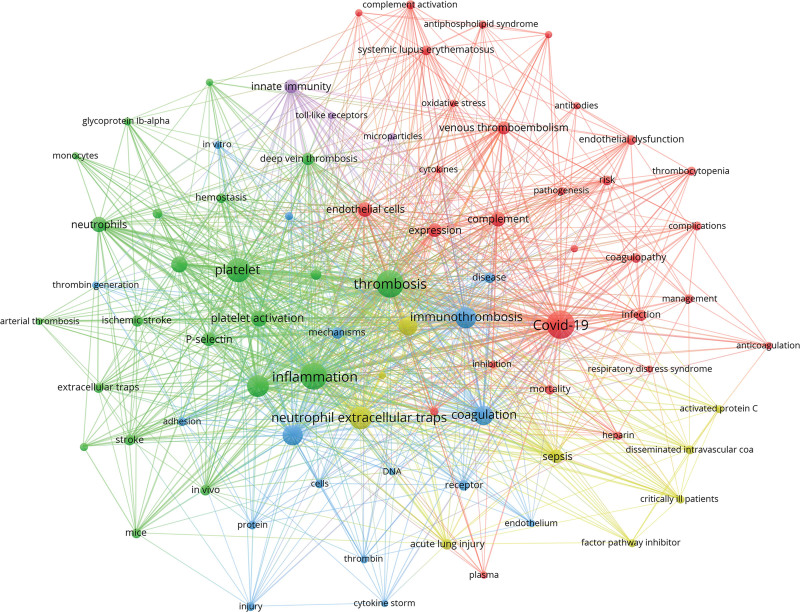
Network visualization of author keywords. The color of the nodes represents the clusters to which the keywords belong, the size of the nodes represents the frequency of keyword occurrence, and the thickness of the lines connecting nodes indicates how frequently different keywords appear together in the same paper.

This study conducted an analysis of the author keywords in 495 target papers, selecting keywords that appeared no fewer than 10 times (a total of 80). A keyword co-occurrence knowledge map was generated, as shown in Figure 6. Detailed information about keyword clusters can be found in Table 5, and information on the top 20 high-frequency keywords, ranked by occurrence frequency, is provided in Table 6.

**Table 5:** Cluster information for keywords.

Cluster	Cluster color	Topic	Keywords
1	Purple	The composition and mechanism of the intravascular innate immune system	Innate immunity, Toll-like receptors, microparticles.
2	Yellow	Mechanism of neutrophil extracellular trap inducing immunothrombosis in immune system diseases	Neutrophil extracellular traps (NETs), tissue factor (TF), sepsis, acute lung injury, disseminated intravascular coagulation (DIC), activated protein C, critically ill patients, factor pathway inhibitor, nuclear factor kappa-B (NF-κB).
3	Green	Immunothrombosis in cardiovascular and cerebrovascular diseases	Thrombosis, inflammation, platelet, thromboinflammation, platelet activation, Von Willebrand factor (vWF), neutrophils, P-selectin, deep vein thrombosis, ischemic stroke, in vivo, stroke, venous thrombosis, extracellular traps, atherosclerosis, hemostasis, mice, acute ischemic stroke, glycoprotein Ib-alpha (GP1BA), arterial thrombosis, monocytes, myocardial infarction.
4	Red	Immunothrombosis in COVID-19	COVID-19, complement, endothelial cells, expression, venous thromboembolism, infection, risk, coagulopathy, endothelial dysfunction, mortality, systemic lupus erythematosus, complement activation, complications, management, thrombocytopenia, antiphospholipid syndrome, heparin, plasminogen activator inhibitor-1, pathogenesis, antiphospholipid antibodies, cytokines, inhibition, thromboembolism, antibodies, anticoagulation, oxidative stress, plasma, respiratory distress syndrome, risk factors.
5	Blue	Molecular mechanism of immunothrombosis	Immunothrombosis, activation, coagulation, tissue factor expression, mechanisms, disease, receptor, adhesion, cells, injury, protein, in vitro, thrombin, DNA, cytokine storm, endothelium, thrombin generation.

**Table 6:** High-frequency keywords.

Rank	Keyword	Occurrences	Rank	Keyword	Occurrences
1	COVID-19	161	11	Platelet activation	51
2	Thrombosis	152	12	vWF	50
3	Inflammation	144	13	Neutrophils	48
4	Platelet	120	14	Complement	43
5	NETs	103	15	Endothelial cells	41
6	Thromboinflammation	97	16	Expression	38
7	Immunothrombosis	90	17	Innate immunity	38
8	Activation	83	18	Sepsis	38
9	Coagulation	77	19	P-selectin	35
10	TF	68	20	Venous thromboembolism	31

High-frequency keywords summarize the research focuses in the field of immunothrombosis. “COVID-19” is the most frequently appearing keyword. “Thrombus formation” and “inflammation” are the physiological processes of immunothrombosis. “Platelets” are the material basis for the process of immunothrombosis. “NETs” represent an important mechanism inducing thrombus formation. “TF,” “ Von Willebrand factor (vWF),” “complement,” and “P-selectin” are key molecules on immunothrombosis process.

**Figure 7.**
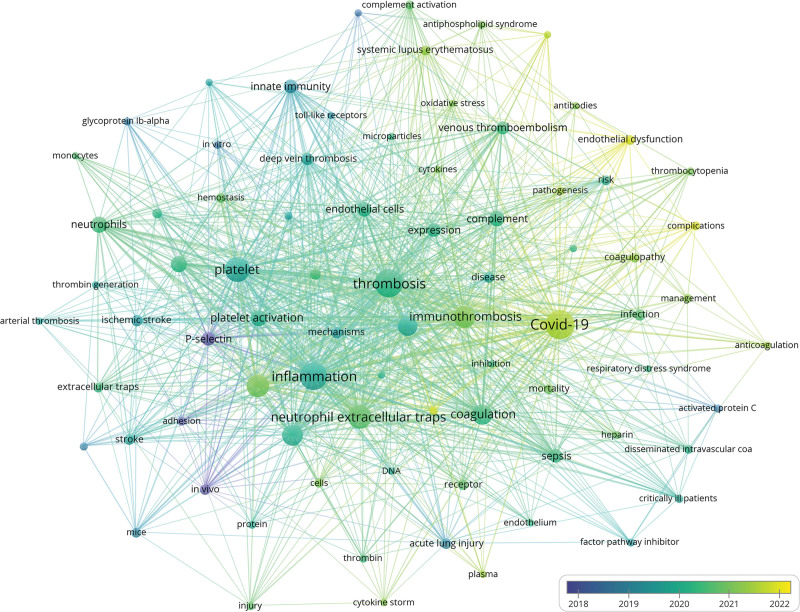
Overlay visualization of keywords average publication year. The size of nodes represents the frequency of keyword occurrence, the thickness of the lines connecting nodes indicates how frequently different keywords appear together in the same paper, and the color of nodes represents the average publication year of the keyword.

Analyzing the average publication years of keywords provides insights into the changing trends of research hotspots in the field of immunothrombosis. Figure 7 visualizes the relationship between the frequency of occurrence and the average publication year of these 80 keywords. Among the 80 high-frequency keywords, most have an average publication year around 2020. “P-selectin,” “adhesion,” and “in vivo” appeared earlier (in 2018 or earlier), while “endothelial dysfunction,” “complications,” and “plasminogen activator inhibitor-1 (PAI-1)” appeared later, around 2022, indicating emerging research hotspots.

### 3.6. Hotspots and frontiers

**Figure 8.**
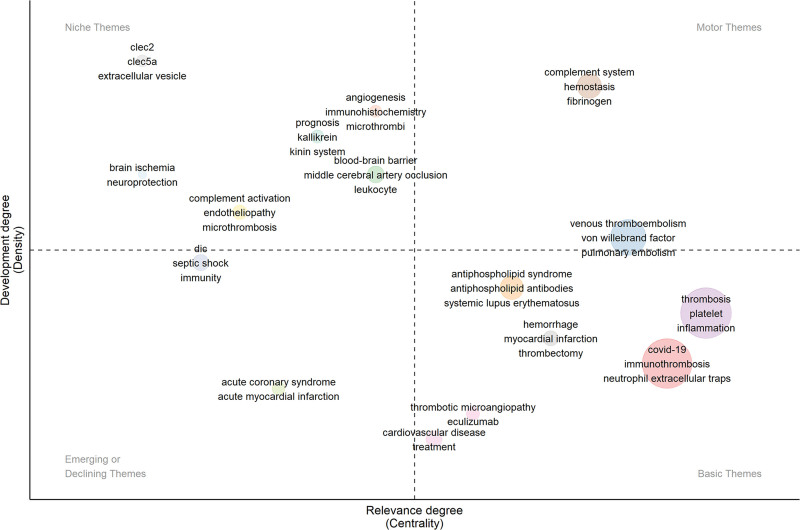
The thematic map of keywords.

A thematic map (Fig. 8) was generated for the keywords, with the horizontal axis representing the relevance to the field and the vertical axis representing the development level of the theme. Thus, research topics represented by various keywords were divided into 4 quadrants.

*Motor themes*: These represent research topics with high relevance to the field and rapid development. In recent years, “complement system,” “vWF,” and “fibrinogen” have become key in exploring the mechanisms of immunothrombosis. The relationship between immunothrombosis and processes like “hemostasis,” “VET,” and “pulmonary embolism (PE)” is a major research focus.

*Basic themes*: These indicate topics with lower development but high relevance, serving as the foundation of research in the field. Research related to “thrombosis,” “platelet,” “inflammation,” and “NETs” is fundamental, exploring the processes and mechanisms of immunothrombosis. Under the “COVID-19” theme, studies analyze the connection between the coagulation system and the immune system after COVID-19 infection from the perspective of immunothrombosis.

*Niche themes*: These refer to low-relevance but highly developing niche topics. “Blood-brain barrier,” “middle cerebral artery,” “leukocyte,” “immunohistochemistry,” “microthrombi,” “endotheliopathy,” “microthrombosis,” and “extracellular vesicle” are relatively novel topics with fewer associated papers.

*Emerging or declining themes*: The topics in this quadrant represent either newly discovered or declining themes. There are 5 themes in this quadrant, these topics have seen fewer explorations in recent years.

**Figure 9.**
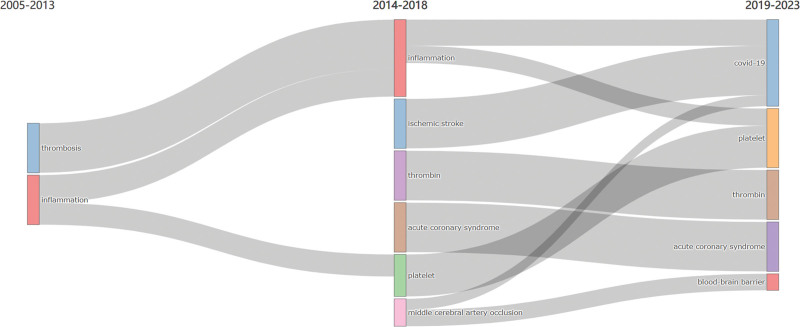
Sankey diagram of keywords evolution.

Considering that the concept of “immunothrombosis” was first defined in papers published in 2013, and in light of the widespread outbreak of COVID-19 in 2019, leading to an in-depth investigation of the connection between COVID-19 and immunothrombosis, three-time intervals, namely 2005 to 2013, 2014 to 2018, and 2019 to 2023, were chosen to generate keywords Sankey diagram (Fig. 9).

As depicted in Figure 9, before the term “immunothrombosis” emerged, the research focus in this field was on “thrombosis” and “inflammation.” From 2014 to 2018, the emphasis shifted towards “inflammation,” and new keywords such as “ischemic stroke,” “acute coronary syndrome,” and “middle cerebral artery occlusion” were introduced. This period reflected researchers’ recognition of the role of immunothrombosis in cardiovascular and cerebrovascular diseases. Additionally, the appearance of “thrombin” and “platelet” indicated a growing interest in understanding the mechanisms of immunothrombosis.

## 4. Discussion

### 4.1. General information

The annual publication volume in the field of immunothrombosis reflects the progress of this domain. Despite the search data covering the period from 2003 to 2023, there were no reported studies before 2005. From 2005 to 2013, the average annual publication volume in the field of immunothrombosis was 1.6 papers, with a total of 14 publications, indicating a relatively sparse research landscape in this area. The earliest published study discovered that platelet was an essential role in the cascade of inflammatory reactions.^[39]^ During this period, research focused more on the interaction between platelets and leukocytes, with platelet-expressed Toll-like receptor (TLR) 2 and P-selectin participating in the processes of thrombosis and/or inflammatory reactions.^[2,40]^ On the other hand, the binding of endothelial cell E-selectin to neutrophils led to the capture of platelets, causing vascular damage.^[41]^ Before the concept of “immunothrombosis” was defined, researchers described this phenomenon as “Thromboinflammation” or “Thromboinflammatory.” From 2014 to 2018, there was a significant increase in the number of publications in this field, with an average annual publication volume of 14.2 papers, indicating that the confirmation of the concept stimulated the development of related research in the field. In 2019, the annual publication volume was 44 papers, whereas the average annual publication volume increased to 91.5 papers after 2020. It is worth noting that among the 366 target publications published since 2020, a total of 289 papers are related to immune dysregulation and thrombosis, of which 180 papers focusing specifically on COVID-19, accounting for 62% (for more details, see Table S2, Supplemental Digital Content, http://links.lww.com/MD/N523). This indicates that a surge in annual publications of thrombosis application is closely related to the prevalence of COVID-19.

The collaboration among core authors reflects the level of activity among researchers in the field, and the formation of research teams also indicates the vitality of the field. All high-productivity authors in Table 1 belong to the large research group in Figure 2B. This suggests that close collaboration among core authors may promote the publication of articles and enhance research quality, accelerating the development of the field of immunothrombosis. Figure 2B showed that new research groups are gradually emerging and developing under the experienced researchers such as Steffen Massberg and John D Lambris. Additionally, among the 25 independent research teams, 14 (64%) have an average publication time later than 2019. All these factors indicate that research on this field is in a continuous upward trend of development.

In our analysis of high-productivity authors and institutions, we found that establishing collaboration between countries and institutions is beneficial for increasing both the quantity and quality of research papers. Countries such as the United States, the United Kingdom, and Germany not only hold the top 3 positions in terms of the number of papers but also maintain close international collaboration relationships between nations and institutions affiliated with these high-productivity Western countries. It is noteworthy that Harvard Medical School has achieved high citation numbers through multiple involvements in high-quality research rather than leading the research itself.^[8]^

The journals publishing articles related to immunothrombosis are all classified as Journal Impact Factor Quartile 1 journals, meaning that publications related to immunothrombosis have been recognized by mainstream academic journals. Interestingly, there is an association between the proportion of open-access (OA) articles in journals and their publication and citation volumes. OA journals such as *Frontiers in Immunology* and *International Journal of Molecular Sciences* contribute significantly to the publication of articles. On the other hand, traditional subscription-based journals like the *Journal of Thrombosis and Haemostasis* and *Blood* have higher average citations per paper. The publication volume of OA journals is notably higher than that of non-OA journals, suggesting that OA journals have contributed to the dissemination of research outcomes in the field of immunothrombosis and facilitated equitable access to knowledge. Traditional journals, meanwhile, have contributed relatively more high-quality research.

### 4.2. Keywords cluster

#### 4.2.1. Cluster 1: the intravascular innate immune system

Cluster 1 encompasses research content that can be summarized as the composition and functional mechanisms of the intravascular innate immune system, emphasizing the significance of platelet pattern recognition receptors. The intravascular innate immune system consists of blood cascade systems (complement, coagulation, and fibrinolysis systems), blood cells (leukocytes, platelets), and endothelial cells. This intrinsic intravascular immune system mediates immune responses and thrombosis to purify the blood. Essentially, it protects the body, but dysregulation of the intravascular innate immune system leads to thromboinflammation.^[5]^

Platelets play a crucial role in preventing inflammation-related bleeding and protecting tissue cells from damage. Platelet-leukocyte interactions are at the core of intravascular innate immunity and contribute to thrombosis.^[42]^ The TLRs on the surface of platelet membranes play essential roles in mediating platelet aggregation, platelet-leukocyte binding, NETs activation, and thrombin formation. TLR4, TLR1/2, and TLR9 can activate downstream signaling pathways through the PI3K/Akt pathway, participating in immunothrombosis.^[43]^ Additionally, platelet-derived extracellular vesicles play a crucial role in recruiting leukocytes and facilitating intravascular immunothrombosis. Platelet-derived extracellular vesicles can serve as biomarkers for disease progression and severity.^[44]^

Furthermore, the complement system is an integral part of the intravascular innate immune system, serving as its backbone. Coagulation factors in the blood, including thrombin and FXa, can cleave complement Component 5 (C5), leading to the formation of complement C3a and C5a, as well as the membrane attack complex C5b-9. This process results in cell lysis and death.^[45]^

#### 4.2.2. Cluster 2: the relationship between NETs and immunothrombosis

In Cluster 2, the relevant research topics of the keywords includes mechanisms by which NETs induce immunothrombosis in different diseases and therapeutic targets related to NETs. NETs are DNA mesh structures modified with histones and granule proteins, representing a unique link between inflammation and thrombosis. Overall, the histones in the structure of NETs can stimulate platelet aggregation, while providing a scaffold for the adhesion of red blood cells. Simultaneously, NETs capture platelet adhesion molecules (vWF, fibrinogen, fibronectin) from the plasma, supporting the formation of thrombin-dependent fibrin, thereby stabilizing thrombosis.^[46]^

In cardiovascular diseases, neutrophils aggregate in the vascular lesions, releasing NETs to promote the intravascular thrombosis.^[47]^ In this process, key roles are played by peptidylarginine deiminase (PAD) 4, vWF, platelet TLR4, P-selectin, high mobility group protein 1, neutrophil solute carrier family 44 member 2, and TF.^[48]^

In COVID-19, the live COVID-19 virus can stimulate the release of NETs from human neutrophils in a dose-dependent manner through PAD4-mediated citrullination.^[49]^ Simultaneously, COVID-19 triggers the activation of the complement system, where complement C3 activates platelets, NETs, and TF expression.^[4]^ Therefore, there is a strong correlation between NETs and the severity of COVID-19 respiratory diseases.^[50,51]^ The combined action of complement, platelets, NETs, and the TF/thrombin axis contributes to the formation and exacerbation of immunothrombosis within the pulmonary and renal microcirculation of patients, leading to organ failure.

In systemic lupus erythematosus, regulated in development and DNA damage responses 1 regulates autophagy-driven NETs formation, enriching NETs with TF and interleukin (IL) 17A, which are crucial factors in promoting immunothrombosis and tissue fibrosis, respectively.^[12]^ In sepsis, lipopolysaccharide induces the activation of PAD and the formation of NETs mediated by the PAD-NETs-citrullinated histone H3 pathway. Additionally, lipopolysaccharide activates platelets in a platelet TLR4-dependent manner, promoting thrombin production and leading to immunothrombosis.^[52]^

Targeting the characteristics of NETs in immunothrombosis, corresponding treatment strategies include targeting thromboregulatory protein,^[53]^ targeting the orosomucoid 1 gene,^[54]^ inhibiting vWF,^[55]^ inhibiting DNA degradation and the complement system,^[56]^ inhibiting PAD4,^[50]^ and using Fostamatinib.^[57]^

#### 4.2.3. Cluster 3: immunothrombosis in cardiovascular and cerebrovascular diseases

Cluster 3 primarily focuses on the connection between inflammatory responses and thrombosis. Targeting immunothrombosis in diseases such as VET, atherosclerosis and stroke is a novel approach for treating these thromboembolic disorders. The interaction between platelets and leukocytes identified as a crucial link in triggering thrombotic diseases.^[58]^

In venous thrombosis, platelets play a more significant role in recruiting and activating neutrophils and less as hemostatic cells. Platelets recruit neutrophils to the site of inflammation, participating in the initiation of venous thrombus formation and leading to NETs-induced immunothrombosis.^[59,60]^ In arterial thrombosis, T helper type 1 cells promote the formation of atherosclerotic plaques, and the destabilization and rupture of these plaques activate platelets. Simultaneously, TF secreted by immune cells triggers a cascade of coagulation reactions, ultimately resulting in arterial thrombosis.^[61]^ Additionally, platelet-derived Gremlin-1 acts as an inflammatory mediator, inducing acute coronary syndrome.^[62]^

For immunothrombosis in venous thrombosis, promising therapeutic targets include proline-rich tyrosine kinase 2,^[63]^ enhancing CD39 expression,^[64]^ targeting the Grb2-associated binder 2/mucosa-associated lymphoid tissue lymphoma translocation protein 1 axis with mucosa-associated lymphoid tissue lymphoma translocation protein 1 inhibitors.^[65]^ In the context of immunothrombosis in arterial thrombosis, potential effective strategies involve targeting P-selectin,^[66]^ inhibiting FXIIa,^[67]^ inducing histamine deficiency,^[68]^ regulating the annexin A1/formyl peptide receptor 2/ALX (AnxA1/FPR2/ALX pathway),^[37]^ interferon λ1/IL-29 treatment,^[69]^ and inhibiting Rho-associated coiled-coil protein kinase.^[70]^

Additionally, targeting CD39,^[71]^ the NETs-vWF axis,^[55,72]^ PAD4, and NADPH oxidase,^[36]^ inhibiting CD147,^[73]^ and suppressing plasma kallikrein all have inhibitory effects on the formation of stroke.^[74]^

#### 4.2.4. Cluster 4: immunothrombosis in COVID-19

The terms in Cluster 4 are all related to the connection between COVID-19 and immunothrombosis, covering research directions such as the pathological mechanisms, clinical biomarkers and potential therapeutic targets of immunothrombosis in COVID-19. Distinct from general pneumonia, vascular neutrophil recruitment, NETs, and immunothrombosis are typical features of COVID-19.^[75]^ In some COVID-19 patients, PE may primarily result from locally induced immunothrombosis by COVID-19 rather than deep venous thrombosis.^[76]^ COVID-19 alters the transcription of platelets, promoting thrombosis.^[77]^ On the other hand, COVID-19 shifts the gene expression profile of monocytes from a typical innate immune response to a pro-thrombotic characteristic, making them highly sensitive to platelets and facilitating platelet aggregation and thrombosis.^[78]^ COVID-19 induces immunothrombosis through NETs, endothelial dysfunction induction,^[79]^ promoting TF-mediated inflammatory responses, and coagulation.^[80]^ Considering the characteristics of immunothrombosis in COVID-19, potential therapeutic targets include the C-type lectin member 5A and TLR2,^[81]^ antithrombin,^[82]^ C-C motif chemokine ligand 2,^[83]^ the complement system, NETs,^[4]^ and IL-6.^[84]^ Based on these targets, potential drugs for treating critically ill COVID-19 patients include complement inhibitors,^[85]^ kininase/kinin inhibitors,^[86]^ α2-macroglobulin,^[87]^ PAD4 inhibitors,^[88]^ and activated protein C analogs.^[89]^ Additionally, biomarkers such as receptor for advanced glycation end products, SARS-CoV-2 nucleocapsid antigen, IL-6, IL-10, tumor necrosis factor receptor-1,^[90]^ IL-4,^[91]^ D-dimer/endogenous thrombin potential ratio,^[92]^ neutrophil-to-platelet ratio,^[93]^ clot lysis time, soluble thrombomodulin, plasminogen activator inhibitor-1, and plasminogen can be used to predict severe COVID-19.^[94]^

#### 4.2.5. Cluster 5: molecular mechanism of immunothrombosis

Cluster 5 illustrates the detailed mechanisms through which immunothrombosis induces various immune system diseases or contributes to disease development. Within the innate immune system, the blood vessels ensure their structural integrity and protection against pathogen invasion through coagulation. TF is a crucial initiator of the coagulation cascade, and the clot composed of fibrin serves as the scaffold for immunothrombosis. Erythrocytes act as bait to attract pathogens, while platelets, by initiating the complement system, releasing chemotactic factors, and expressing P-selectin, recruit leukocytes, activate FXII, and induce NETs, linking inflammation and coagulation. This process guides immune cells to the infected sites within the circulation.^[1]^ It is noteworthy that TF plays a central role in triggering the process of immunothrombosis. Innate immune signals induce the expression of the TF gene in immune cells through NF-κB and activate stimulator of interferon genes and inflammasomes to trigger TF release,^[95]^ driving the coagulation cascade.^[96]^ Therefore, targeting TF is currently a widely studied therapeutic strategy.

APS is a typical immunothrombosis disorder. In vitro studies have found that antiphospholipid antibodies can promote the expression of TF, induce the release of pro-inflammatory cytokines, and trigger immunothrombosis by inducing NETs.^[31]^

In sepsis, pathogens directly activate endothelial cells and platelets through their pathogen-associated molecular patterns, triggering NETs and leading to uncontrolled immunothrombosis in the microcirculation.^[11]^

In acute respiratory distress syndrome, accompanying lung damage, platelets promote the recruitment of immune cells by expressing intracellular adhesion molecule-1, vascular cell adhesion molecule-1, and P-selectin. The formation of platelet–neutrophil complexes activates triggering receptors expressed on myeloid cells-1, leading to the release of a large amount of pro-inflammatory cytokines and chemokines, further inducing immunothrombosis.^[13]^

In respiratory diseases associated with RNA virus infections, immunothrombosis may be based on a positive feedback mechanism. Thrombin, through the activation of protease-activated receptor 1/2, maintains and amplifies the pro-thrombotic and pro-inflammatory effects exposed to viral RNA mimics like poly(I:C) on endothelial cells.^[97]^

### 4.3. Further development

The overall trend in the research topics related to immunothrombosis is illustrated in Figure 9. Over the past 20 years, researchers initially observed the correlation between thrombosis and inflammatory reactions in clinical cases, recognizing the crucial role of platelets in the immune system and the involvement of leukocytes in the process of thrombosis. After the introduction of the concept of “immunothrombosis,” researchers shifted their focus to the role of immunothrombosis in cardiovascular and cerebrovascular diseases.

However, with the outbreak of COVID-19 in 2019, thromboembolic events in COVID-19 patients garnered unprecedented attention from researchers. Future research directions for researchers may be guided by the results in Figure 8. The primary research focus continues to center on the formation of immunothrombosis in COVID-19. In-depth molecular mechanism studies can still be conducted on NETs and platelets as crucial components of immunothrombosis. Additionally, the complement system, vWF, and fibrinogen are expected to become new hotspots for in-depth research, as their roles in immunothrombosis are not yet fully understood. In terms of related diseases, targeting immunothrombosis and developing clinical drug strategies for diseases such as COVID-19, APS, PE, coronary artery disease, and VET will be crucial research directions in the future. Besides, topics within niche themes may provide researchers with more perspectives. Research directions such as brain ischemia, middle cerebral artery occlusion, and neuroprotection are worthy of attention.^[58,98,99]^

### 4.4. Limitations

One limitation of this study is that all data were collected solely from WOSCC, potentially omitting articles from other databases such as PubMed and Scopus. Additionally, the number of published articles in the field of immunothrombosis increased significantly in 2023, with new research findings emerging rapidly. During the course of this study, the latest publications in this field were not included for analysis. These factors contribute to incomplete data collection, which may introduce certain limitations to the findings presented in this paper.

## 5. Conclusions

Immunothrombosis is a physiological process of mutual influence between the immune system and the coagulation system. It represents a form of innate immunity within blood vessels. This study employs bibliometric methods to analyze the development and frontier research topics in the field of immunothrombosis over the past 20 years. The number of research papers in this field is growing rapidly, with emerging research groups continuously contributing.

Research focus in the field of immunothrombosis has evolved from the initial study of the interaction between platelets and leukocytes to the current exploration of key components such as NETs, the complement system, and TF. Inhibiting inflammatory reactions has emerged as a potential new strategy for treating thromboembolic diseases. On the other hand, achieving a balance between immune responses and coagulation during disease treatment and modulating the extent of immunothrombosis have become critical considerations. Finding treatment targets that are more specific and controllable presents one of the current challenges in this field.

## Author contributions

**Conceptualization:** Mengyu Hou, Ruihua Dong.

**Data curation:** Mengyu Hou, Jingxuan Wu.

**Formal analysis:** Jiangshuo Li, Meijuan Zhang, Hang Yin.

**Funding acquisition:** Jingxuan Wu, Ruihua Dong.

**Methodology:** Mengyu Hou.

**Supervision:** Ruihua Dong.

**Visualization:** Mengyu Hou, Jingxuan Wu.

**Writing – original draft:** Jingcheng Chen, Zhili Jin.

**Writing – review & editing:** Ruihua Dong.

## Supplementary Material



## References

[1] Gaertner F Massberg S. Blood coagulation in immunothrombosis-at the frontline of intravascular immunity. Semin Immunol. 2016;28:561–9. 27866916 10.1016/j.smim.2016.10.010

[2] Rondina MT Weyrich AS Zimmerman GA. Platelets as cellular effectors of inflammation in vascular diseases. Circ Res. 2013;112:1506–19. 23704217 10.1161/CIRCRESAHA.113.300512 PMC3738064

[3] Cesta MC Zippoli M Marsiglia C. Neutrophil activation and neutrophil extracellular traps (NETs) in COVID-19 ARDS and immunothrombosis. Eur J Immunol. 2023;53:e2250010. 36239164 10.1002/eji.202250010 PMC9874644

[4] Skendros P Mitsios A Chrysanthopoulou A. Complement and tissue factor-enriched neutrophil extracellular traps are key drivers in COVID-19 immunothrombosis. J Clin Invest. 2020;130:6151–7. 32759504 10.1172/JCI141374 PMC7598040

[5] Ekdahl KN Teramura Y Hamad OA. Dangerous liaisons: complement, coagulation, and kallikrein/kinin cross-talk act as a linchpin in the events leading to thromboinflammation. Immunol Rev. 2016;274:245–69. 27782319 10.1111/imr.12471

[6] Schrottmaier WC Mussbacher M Salzmann M Assinger A. Platelet-leukocyte interplay during vascular disease. Atherosclerosis. 2020;307:109–20. 32439204 10.1016/j.atherosclerosis.2020.04.018

[7] Colling ME Tourdot BE Kanthi Y. Inflammation, infection and venous thromboembolism. Circ Res. 2021;128:2017–36. 34110909 10.1161/CIRCRESAHA.121.318225 PMC8202069

[8] Gupta A Madhavan MV Sehgal K. Extrapulmonary manifestations of COVID-19. Nat Med. 2020;26:1017–32. 32651579 10.1038/s41591-020-0968-3 PMC11972613

[9] Li S Wang H Shao Q. The central role of neutrophil extracellular traps (NETs) and by-products in COVID-19 related pulmonary thrombosis. Immun Inflamm Dis. 2023;11:e949. 37647446 10.1002/iid3.949 PMC10461423

[10] Engelmann B Massberg S. Thrombosis as an intravascular effector of innate immunity. Nat Rev Immunol. 2013;13:34–45. 23222502 10.1038/nri3345

[11] Iba T Levi M Levy JH. Intracellular communication and immunothrombosis in sepsis. J Thromb Haemost. 2022;20:2475–84. 35979601 10.1111/jth.15852 PMC9804233

[12] Frangou E Chrysanthopoulou A Mitsios A. REDD1/autophagy pathway promotes thromboinflammation and fibrosis in human systemic lupus erythematosus (SLE) through NETs decorated with tissue factor (TF) and interleukin-17A (IL-17A). Ann Rheum Dis. 2019;78:238–48. 30563869 10.1136/annrheumdis-2018-213181 PMC6352428

[13] Frantzeskaki F Armaganidis A Orfanos SE. Immunothrombosis in acute respiratory distress syndrome: cross talks between Inflammation and coagulation. Respiration. 2017;93:212–25. 27997925 10.1159/000453002

[14] Szepanowski RD Haupeltshofer S Vonhof SE Frank B Kleinschnitz C Casas AI. Thromboinflammatory challenges in stroke pathophysiology. Semin Immunopathol. 2023;45:389–410. 37273022 10.1007/s00281-023-00994-4 PMC10241149

[15] Stark K Massberg S. Interplay between inflammation and thrombosis in cardiovascular pathology. Nat Rev Cardiol. 2021;18:666–82. 33958774 10.1038/s41569-021-00552-1 PMC8100938

[16] Cebo M Dittrich K Fu X. Platelet ACKR3/CXCR7 favors antiplatelet lipids over an atherothrombotic lipidome and regulates thromboinflammation. Blood. 2022;139:1722–42. 34905596 10.1182/blood.2021013097

[17] Bonaventura A Vecchié A Dagna L. Endothelial dysfunction and immunothrombosis as key pathogenic mechanisms in COVID-19. Nat Rev Immunol. 2021;21:319–29. 33824483 10.1038/s41577-021-00536-9 PMC8023349

[18] Musgrave KM Scott J Sendama W. Tissue factor expression in monocyte subsets during human immunothrombosis, endotoxemia and sepsis. Thromb Res. 2023;228:10–20. 37263122 10.1016/j.thromres.2023.05.018

[19] Ninkov A Frank JR Maggio LA. Bibliometrics: methods for studying academic publishing. Perspect Med Educ. 2022;11:173–6. 34914027 10.1007/s40037-021-00695-4 PMC9240160

[20] Song Y Wei K Yang S Shu F Qiu J. Analysis on the research progress of library and information science since the new century. Libr Hi Tech. 2023;41:1145–57.

[21] Sun HL Bai W Li XH. Schizophrenia and inflammation research: a bibliometric analysis. Front Immunol. 2022;13:907851. 35757702 10.3389/fimmu.2022.907851 PMC9219580

[22] Van Eck NJ Waltman L. Software survey: VOSviewer, a computer program for bibliometric mapping. Scientometrics. 2010;84:523–38. 20585380 10.1007/s11192-009-0146-3 PMC2883932

[23] Aria M Cuccurullo C. bibliometrix: an R-Tool for comprehensive science mapping analysis. J Informetr. 2018;11:959–75.

[24] Price DJDS Merton RK Garfield E. Little Science, Big Science...and Beyond. New York, NY: Columbia University Press; 1986.

[25] Storm BS Christiansen D Fure H. Air bubbles activate complement and trigger hemostasis and C3-dependent cytokine release ex vivo in human whole blood. J Immunol. 2021;207:2828–40. 34732467 10.4049/jimmunol.2100308 PMC8611197

[26] Storm BS Ludviksen JK Christiansen D. Venous air embolism activates complement C3 without corresponding C5 activation and trigger thromboinflammation in pigs. Front Immunol. 2022;13:839632. 35371063 10.3389/fimmu.2022.839632 PMC8964959

[27] Ortega-Paz L Talasaz AH Sadeghipour P. COVID-19-associated pulmonary embolism: review of the pathophysiology, epidemiology, prevention, diagnosis, and treatment. Semin Thromb Hemost. 2023;49:816–32. 36223804 10.1055/s-0042-1757634

[28] Bikdeli B Talasaz AH Rashidi F. Intermediate versus standard-dose prophylactic anticoagulation and statin therapy versus placebo in critically-ill patients with COVID-19: rationale and design of the INSPIRATION/INSPIRATION-S studies. Thromb Res. 2020;196:382–94. 32992075 10.1016/j.thromres.2020.09.027 PMC7513771

[29] Bikdeli B Madhavan MV Gupta A. Pharmacological agents targeting thromboinflammation in COVID-19: review and implications for future research. Thromb Haemost. 2020;120:1004–24. 32473596 10.1055/s-0040-1713152 PMC7516364

[30] Madison JA Gockman K Hoy C Tambralli A Zuo Y Knight JS. Pediatric antiphospholipid syndrome: clinical features and therapeutic interventions in a single center retrospective case series. Pediatr Rheumatol Online J. 2022;20:17. 35197077 10.1186/s12969-022-00677-8 PMC8867616

[31] Knight JS Kanthi Y. Mechanisms of immunothrombosis and vasculopathy in antiphospholipid syndrome. Semin Immunopathol. 2022;44:347–62. 35122116 10.1007/s00281-022-00916-w PMC8816310

[32] Ambati A Knight JS Zuo Y. Antiphospholipid syndrome management: a 2023 update and practical algorithm-based approach. Curr Opin Rheumatol. 2023;35:149–60. 36866678 10.1097/BOR.0000000000000932 PMC10364614

[33] Harrison SR Klassen JRL Bridgewood C Scarsbrook A Marzo-Ortega H McGonagle D. Chest pain mimicking pulmonary embolism may be a common presentation of COVID-19 in ambulant patients without other typical features of infection. J Intern Med. 2021;290:349–58. 33560545 10.1111/joim.13267 PMC8013761

[34] McGonagle D Ramanan AV Bridgewood C. Immune cartography of macrophage activation syndrome in the COVID-19 era. Nat Rev Rheumatol. 2021;17:145–57. 33547426 10.1038/s41584-020-00571-1 PMC7863615

[35] McGonagle D Kearney MF O’Regan A. Therapeutic implications of ongoing alveolar viral replication in COVID-19. Lancet Rheumatol. 2022;4:e135–44. 34873587 10.1016/S2665-9913(21)00322-2 PMC8635460

[36] Ansari J Vital SA Yadav S Gavins FNE. Regulating neutrophil PAD4/NOX-dependent cerebrovasular thromboinflammation. Int J Biol Sci. 2023;19:852–64. 36778112 10.7150/ijbs.77434 PMC9910005

[37] Ansari J Senchenkova EY Vital SA. Targeting the AnxA1/Fpr2/ALX pathway regulates neutrophil function, promoting thromboinflammation resolution in sickle cell disease. Blood. 2021;137:1538–49. 33512489 10.1182/blood.2020009166 PMC7976506

[38] Senchenkova EY Ansari J Becker F. Novel role for the AnxA1-Fpr2/ALX signaling axis as a key regulator of platelet function to promote resolution of inflammation. Circulation. 2019;140:319–35. 31154815 10.1161/CIRCULATIONAHA.118.039345 PMC6687438

[39] Pasupathy S Naseem KM Homer-Vanniasinkam S. Effects of warm-up on exercise capacity, platelet activation and platelet-leucocyte aggregation in patients with claudication. Br J Surg. 2005;92:50–5. 15505876 10.1002/bjs.4798

[40] Wacker J Lucchinetti E Jamnicki M. Delayed inhibition of agonist-induced granulocyte-platelet aggregation after low-dose sevoflurane inhalation in humans. Anesth Analg. 2008;106:1749–58. 18499605 10.1213/ane.0b013e318172f9e9

[41] Hidalgo A Chang J Jang JE Peired AJ Chiang EY Frenette PS. Heterotypic interactions enabled by polarized neutrophil microdomains mediate thromboinflammatory injury. Nat Med. 2009;15:384–91. 19305412 10.1038/nm.1939 PMC2772164

[42] Dib PRB Quirino-Teixeira AC Merij LB. Innate immune receptors in platelets and platelet-leukocyte interactions. J Leukoc Biol. 2020;108:1157–82. 32779243 10.1002/JLB.4MR0620-701R

[43] D' Atri LP Schattner M. Platelet toll-like receptors in thromboinflammation. Front Biosci (Landmark Ed). 2017;22:1867–83. 28410150 10.2741/4576

[44] Goubran H Seghatchian J Sabry W Ragab G Burnouf T. Platelet and extracellular vesicles in COVID-19 infection and its vaccines. Transfus Apher Sci. 2022;61:103459. 35654711 10.1016/j.transci.2022.103459 PMC9122775

[45] Keragala CB Draxler DF McQuilten ZK Medcalf RL. Haemostasis and innate immunity-a complementary relationship: a review of the intricate relationship between coagulation and complement pathways. Br J Haematol. 2018;180:782–98. 29265338 10.1111/bjh.15062

[46] Fuchs TA Brill A Duerschmied D. Extracellular DNA traps promote thrombosis. Proc Natl Acad Sci U S A. 2010;107:15880–5. 20798043 10.1073/pnas.1005743107 PMC2936604

[47] Shirakawa K Sano M. Neutrophils and neutrophil extracellular traps in cardiovascular disease: an overview and potential therapeutic approaches. Biomedicines. 2022;10:1850. 36009397 10.3390/biomedicines10081850 PMC9405087

[48] Van Bruggen S Martinod K. The coming of age of neutrophil extracellular traps in thrombosis: where are we now and where are we headed? Immunol Rev. 2023;314:376–98. 36560865 10.1111/imr.13179

[49] Ackermann M Anders HJ Bilyy R. Patients with COVID-19: in the dark-NETs of neutrophils. Cell Death Differ. 2021;28:3125–39. 34031543 10.1038/s41418-021-00805-z PMC8142290

[50] Middleton EA He XY Denorme F. Neutrophil extracellular traps contribute to immunothrombosis in COVID-19 acute respiratory distress syndrome. Blood. 2020;136:1169–79. 32597954 10.1182/blood.2020007008 PMC7472714

[51] Borczuk AC Yantiss RK. The pathogenesis of coronavirus-19 disease. J Biomed Sci. 2022;29:87. 36289507 10.1186/s12929-022-00872-5 PMC9597981

[52] Chen Z Zhang H Qu M. Review: the emerging role of neutrophil extracellular traps in sepsis and sepsis-associated thrombosis. Front Cell Infect Microbiol. 2021;11:653228. 33816356 10.3389/fcimb.2021.653228 PMC8010653

[53] Helms J Clere-Jehl R Bianchini E. Thrombomodulin favors leukocyte microvesicle fibrinolytic activity, reduces NETosis and prevents septic shock-induced coagulopathy in rats. Ann Intensive Care. 2017;7:118. 29222696 10.1186/s13613-017-0340-z PMC5722785

[54] Lopez S Martinez-Perez A Rodriguez-Rius A. Integrated GWAS and gene expression suggest ORM1 as a potential regulator of plasma levels of cell-free DNA and thrombosis risk. Thromb Haemost. 2022;122:1027–39. 35272364 10.1055/s-0041-1742169 PMC9251712

[55] Yang J Wu Z Long Q. Insights into immunothrombosis: the interplay among neutrophil extracellular trap, von Willebrand Factor, and ADAMTS13. Front Immunol. 2020;11:610696. 33343584 10.3389/fimmu.2020.610696 PMC7738460

[56] Burmeister A Vidal YSS Liu X. Impact of neutrophil extracellular traps on fluid properties, blood flow and complement activation. Front Immunol. 2022;13:1078891. 36591269 10.3389/fimmu.2022.1078891 PMC9800590

[57] Strich JR Ramos-Benitez MJ Randazzo D. Fostamatinib inhibits neutrophils extracellular traps induced by COVID-19 patient plasma: a potential therapeutic. J Infect Dis. 2021;223:981–4. 33367731 10.1093/infdis/jiaa789 PMC7799006

[58] De Meyer SF Langhauser F Haupeltshofer S Kleinschnitz C Casas AI. Thromboinflammation in brain ischemia: recent updates and future perspectives. Stroke. 2022;53:1487–99. 35360931 10.1161/STROKEAHA.122.038733

[59] Heestermans M Poenou G Duchez AC Hamzeh-Cognasse H Bertoletti L Cognasse F. Immunothrombosis and the role of platelets in venous thromboembolic diseases. Int J Mol Sci. 2022;23:13176. 36361963 10.3390/ijms232113176 PMC9656618

[60] Stevens H McFadyen JD. Platelets as central actors in thrombosis-reprising an old role and defining a new character. Semin Thromb Hemost. 2019;45:802–9. 31622994 10.1055/s-0039-1698829

[61] Karbach S Lagrange J Wenzel P. Thromboinflammation and vascular dysfunction. Hamostaseologie. 2019;39:180–7. 30513535 10.1055/s-0038-1676130

[62] Chatterjee M Behrendt A Schmid M. Platelets as a novel source of Gremlin-1: implications for thromboinflammation. Thromb Haemost. 2017;117:311–24. 27929199 10.1160/TH16-08-0665

[63] Momi S Canino J Vismara M. Proline-rich tyrosine kinase Pyk2 regulates deep vein thrombosis. Haematologica. 2022;107:1374–83. 35142150 10.3324/haematol.2021.279703 PMC9152972

[64] Anyanwu AC Kanthi Y Fukase K. Tuning the Thromboinflammatory response to venous flow interruption by the ectonucleotidase CD39. Arterioscler Thromb Vasc Biol. 2019;39:e118–29. 30816804 10.1161/ATVBAHA.119.312407 PMC6467508

[65] Kondreddy V Keshava S Das K Magisetty J Rao LVM Pendurthi UR. The Gab2-MALT1 axis regulates thromboinflammation and deep vein thrombosis. Blood. 2022;140:1549–64. 35895897 10.1182/blood.2022016424 PMC9523376

[66] Pircher J Engelmann B Massberg S Schulz C. Platelet-neutrophil crosstalk in atherothrombosis. Thromb Haemost. 2019;119:1274–82. 31254975 10.1055/s-0039-1692983

[67] Searle AK Chen YC Wallert M. Pharmacological inhibition of factor XIIa attenuates abdominal aortic aneurysm, reduces atherosclerosis, and stabilizes atherosclerotic plaques. Thromb Haemost. 2022;122:196–207. 34619795 10.1055/a-1663-8208 PMC8820844

[68] Li H Tang C Zhu X. Histamine deficiency facilitates coronary microthrombosis after myocardial infarction by increasing neutrophil-platelet interactions. J Cell Mol Med. 2020;24:3504–20. 32064748 10.1111/jcmm.15037 PMC7131923

[69] Chrysanthopoulou A Kambas K Stakos D. Interferon lambda1/IL-29 and inorganic polyphosphate are novel regulators of neutrophil-driven thromboinflammation. J Pathol. 2017;243:111–22. 28678391 10.1002/path.4935

[70] Hsu LW Chen PW Chang WT Lee WH Liu PY. The role of ROCK in platelet-monocyte collaborative induction of thromboinflammation during acute coronary syndrome. Thromb Haemost. 2020;120:1417–31. 32877952 10.1055/s-0040-1714278

[71] Kanthi YM Sutton NR Pinsky DJ. CD39: interface between vascular thrombosis and inflammation. Curr Atheroscler Rep. 2014;16:425. 24838375 10.1007/s11883-014-0425-1 PMC6800993

[72] Denorme F Martinod K Vandenbulcke A. The von Willebrand Factor A1 domain mediates thromboinflammation, aggravating ischemic stroke outcome in mice. Haematologica. 2021;106:819–28. 32107335 10.3324/haematol.2019.241042 PMC7927893

[73] Jin R Xiao AY Chen R Granger DN Li G. Inhibition of CD147 (cluster of differentiation 147) ameliorates acute ischemic stroke in mice by reducing thromboinflammation. Stroke. 2017;48:3356–65. 29114092 10.1161/STROKEAHA.117.018839 PMC5726599

[74] Göb E Reymann S Langhauser F. Blocking of plasma kallikrein ameliorates stroke by reducing thromboinflammation. Ann Neurol. 2015;77:784–803. 25628066 10.1002/ana.24380

[75] Nicolai L Leunig A Brambs S. Vascular neutrophilic inflammation and immunothrombosis distinguish severe COVID-19 from influenza pneumonia. J Thromb Haemost. 2021;19:574–81. 33217134 10.1111/jth.15179 PMC7753335

[76] Franco-Moreno A Herrera-Morueco M Mestre-Gómez B. Incidence of deep venous thrombosis in patients with COVID-19 and pulmonary embolism: compression ultrasound COVID Study. J Ultrasound Med. 2021;40:1411–6. 33017480 10.1002/jum.15524 PMC7675470

[77] Ji W Chen L Yang W. Transcriptional landscape of circulating platelets from patients with COVID-19 reveals key subnetworks and regulators underlying SARS-CoV-2 infection: implications for immunothrombosis. Cell Biosci. 2022;12:15. 35139909 10.1186/s13578-022-00750-5 PMC8827164

[78] Maher AK Burnham KL Jones EM. Transcriptional reprogramming from innate immune functions to a pro-thrombotic signature by monocytes in COVID-19. Nat Commun. 2022;13:7947. 36572683 10.1038/s41467-022-35638-y PMC9791976

[79] De Melo TC Trevisan-Silva D Alvarez-Flores MP. Proteomic analysis identifies molecular players and biological processes specific to SARS-CoV-2 exposure in endothelial cells. Int J Mol Sci. 2022;23:10452. 36142365 10.3390/ijms231810452 PMC9500950

[80] Hottz ED Martins-Gonçalves R Palhinha L. Platelet-monocyte interaction amplifies thromboinflammation through tissue factor signaling in COVID-19. Blood Adv. 2022;6:5085–99. 35420680 10.1182/bloodadvances.2021006680 PMC9015715

[81] Sung PS Yang SP Peng YC Sun CP Tao MH Hsieh SL. CLEC5A and TLR2 are critical in SARS-CoV-2-induced NET formation and lung inflammation. J Biomed Sci. 2022;29:52. 35820906 10.1186/s12929-022-00832-z PMC9277873

[82] Chen-Goodspeed A Dronavalli G Zhang X. Antithrombin activity is associated with persistent thromboinflammation and mortality in patients with severe COVID-19 illness. Acta Haematol. 2023;146:117–24. 36538905 10.1159/000528584 PMC9940263

[83] Nieri D Neri T Barbieri G. C-C motive chemokine ligand 2 and thromboinflammation in COVID-19-associated pneumonia: a retrospective study. Thromb Res. 2021;204:88–94. 34153649 10.1016/j.thromres.2021.06.003 PMC8184876

[84] Mukhopadhyay S Sinha S Mohapatra SK. Analysis of transcriptomic data sets supports the role of IL-6 in NETosis and immunothrombosis in severe COVID-19. BMC Genom Data. 2021;22:49. 34775962 10.1186/s12863-021-01001-1 PMC8590626

[85] Mastellos DC Pires da Silva BGP Fonseca BAL. Complement C3 vs C5 inhibition in severe COVID-19: early clinical findings reveal differential biological efficacy. Clin Immunol. 2020;220:108598. 32961333 10.1016/j.clim.2020.108598 PMC7501834

[86] Lipcsey M Persson B Eriksson O. The outcome of critically Ill COVID-19 patients is linked to thromboinflammation dominated by the kallikrein/kinin system. Front Immunol. 2021;12:627579. 33692801 10.3389/fimmu.2021.627579 PMC7937878

[87] Seitz R Gürtler L Schramm W. Thromboinflammation in COVID-19: can α(2)-macroglobulin help to control the fire? J Thromb Haemost. 2021;19:351–4. 33230947 10.1111/jth.15190 PMC7753444

[88] Elliott W Jr Guda MR Asuthkar S. PAD inhibitors as a potential treatment for SARS-CoV-2 immunothrombosis. Biomedicines. 2021;9:1867. 34944683 10.3390/biomedicines9121867 PMC8698348

[89] Griffin JH Lyden P. COVID-19 hypothesis: activated protein C for therapy of virus-induced pathologic thromboinflammation. Res Pract Thromb Haemost. 2020;4:506–9. 32548551 10.1002/rth2.12362 PMC7292662

[90] Matthay ZA Fields AT Wick KD. Association of SARS-CoV-2 nucleocapsid viral antigen and the receptor for advanced glycation end products with development of severe disease in patients presenting to the emergency department with COVID-19. Front Immunol. 2023;14:1130821. 37026003 10.3389/fimmu.2023.1130821 PMC10070743

[91] Motta Junior JDS Miggiolaro A Nagashima S. Mast cells in alveolar septa of COVID-19 patients: a pathogenic pathway that may link interstitial edema to immunothrombosis. Front Immunol. 2020;11:574862. 33042157 10.3389/fimmu.2020.574862 PMC7530169

[92] De la Morena-Barrio ME Bravo-Pérez C Miñano A. Prognostic value of thrombin generation parameters in hospitalized COVID-19 patients. Sci Rep. 2021;11:7792. 33833254 10.1038/s41598-021-85906-y PMC8032761

[93] López-Escobar A Madurga R Castellano JM. Hemogram as marker of in-hospital mortality in COVID-19. J Investig Med. 2021;69:962–9. 10.1136/jim-2021-001810 33849952

[94] Juneja GK Castelo M Yeh CH. Biomarkers of coagulation, endothelial function, and fibrinolysis in critically ill patients with COVID-19: a single-center prospective longitudinal study. J Thromb Haemost. 2021;19:1546–57. 33826233 10.1111/jth.15327 PMC8250276

[95] Ryan TAJ Preston RJS O’Neill LAJ. Immunothrombosis and the molecular control of tissue factor by pyroptosis: prospects for new anticoagulants. Biochem J. 2022;479:731–50. 35344028 10.1042/BCJ20210522

[96] Ryan TAJ O’Neill LAJ. Innate immune signaling and immunothrombosis: new insights and therapeutic opportunities. Eur J Immunol. 2022;52:1024–34. 35569038 10.1002/eji.202149410 PMC9543829

[97] Subramaniam S Ogoti Y Hernandez I. A thrombin-PAR1/2 feedback loop amplifies thromboinflammatory endothelial responses to the viral RNA analogue poly(I:C). Blood Adv. 2021;5:2760–74. 34242391 10.1182/bloodadvances.2021004360 PMC8288670

[98] Desilles JP Syvannarath V Di Meglio L. Downstream microvascular thrombosis in cortical venules is an early response to proximal cerebral arterial occlusion. J Am Heart Assoc. 2018;7:e007804. 29496683 10.1161/JAHA.117.007804 PMC5866327

[99] Dreikorn M Milacic Z Pavlovic V Meuth SG Kleinschnitz C Kraft P. Immunotherapy of experimental and human stroke with agents approved for multiple sclerosis: a systematic review. Ther Adv Neurol Disord. 2018;11:1756286418770626. 29774055 10.1177/1756286418770626 PMC5949925

