# Supplementary material for: Immunothrombosis: A bibliometric analysis from 2003 to 2023
Source: Medicine (Baltimore). 2024 Sep 13;103(37):e39566. doi: 10.1097/MD.0000000000039566 (PMC11404911; doi:10.1097/MD.0000000000039566)
Supplement: Supplementary file 1 [file medi-103-e39566-s001.docx]

**Article title:** Immunothrombosis: A Bibliometric analysis from 2003 to 2023

**First Author:** Mengyu Hou

**Supplementary Information file 1:**

**Table S1** Detail information about cooperating countries.

| **Table S1** Detail information about cooperating countries | | | | |
| --- | --- | --- | --- | --- |
| Country | Publications | Number of Collaborations | Average Citation | MCP^*^ Ratio (%) |
| USA | 156 | 24 | 53.2 | 23.6 |
| Germany | 88 | 23 | 56.3 | 26.7 |
| UK | 63 | 23 | 34.1 | 37.5 |
| Italy | 43 | 20 | 38.9 | 12.5 |
| China | 41 | 13 | 17.8 | 24.2 |
| Japan | 30 | 8 | 42.6 | 40.9 |
| Canada | 27 | 21 | 31.6 | 23.1 |
| Sweden | 26 | 17 | 34.0 | 66.7 |
| Brazil | 23 | 9 | 24.9 | 20.0 |
| Spain | 23 | 19 | 37.8 | 21.1 |
| France | 23 | 14 | 36.5 | 11.8 |
| Australia | 23 | 16 | 67.3 | 46.7 |
| Netherlands | 21 | 14 | 26.7 | NA |
| Greece | 19 | 19 | 75.4 | 53.8 |
| Belgium | 16 | 13 | 63.7 | 36.4 |
| Norway | 13 | 8 | 7.5 | 70.0 |
| Russia | 10 | 11 | 23.8 | 25.0 |
| Austria | 9 | 11 | 63.4 | 14.3 |
| Poland | 9 | 11 | 21.6 | 16.7 |
| Switzerland | 9 | 14 | 17.3 | 0.0 |
| Ireland | 9 | 6 | 57.2 | 25.0 |
| Denmark | 9 | 15 | 53.2 | 100.0 |
| Mexico | 7 | 2 | 17.4 | 28.6 |
| Iran | 7 | 7 | 27.4 | 40.0 |
| India | 7 | 3 | 16.0 | 50.0 |
| Egypt | 5 | 7 | 3.8 | 100.0 |

^*^MCP: Multiple country publications number.
