# Supplementary material for: Immunothrombosis: A bibliometric analysis from 2003 to 2023
Source: Medicine (Baltimore). 2024 Sep 13;103(37):e39566. doi: 10.1097/MD.0000000000039566 (PMC11404911; doi:10.1097/MD.0000000000039566)
Supplement: Supplementary file 2 [file medi-103-e39566-s002.docx]

**Article title:** Immunothrombosis: A Bibliometric analysis from 2003 to 2023

**First Author:** Mengyu Hou

**Supplementary Information file 2:**

**Table S2** Detail information about publications concentrated on COVID-19.

**Table S2** Detail information about publications concentrated on COVID-19

| No. | Title | Authorship | Keywords | Source | DOI |
| --- | --- | --- | --- | --- | --- |
| 1 | Immunothrombosis: Molecular Aspects and New Therapeutic Perspectives | Marcos-Jubilar M; Lecumberri R; Paramo Ja | Immunothrombosis; Thromboinflammation; Tissue Factor | Journal of Clinical Medicine | 10.3390/jcm12041399 |
| 2 | Thromboinflammation as a Driver of Venous Thromboembolism | Gauchel N; Krauel K; Hamad Ma; Bode C; Duerschmied D | Thromboinflammation; Immunothrombosis; innate Immunity; Venous Thromboembolism; COVID-19 | Hamostaseologie | 10.1055/a-1661-0257 |
| 3 | Dysregulated miRNAs Network in the Critical COVID-19: An Important Clue for Uncontrolled Immunothrombosis/Thromboinflammation | Mortazavi-Jahromi Ss; Aslani M | SARS-Cov-2; COVID-19; Immunothrombosis; miRNAs; miRNAs-Based Therapy | International Immunopharmacology | 10.1016/j.intimp.2022.109040 |
| 4 | Interplay Between Inflammation and Thrombosis in Cardiovascular Pathology | Stark K; Massberg S | NA | Nature Reviews Cardiology | 10.1038/s41569-021-00552-1 |
| 5 | Thromboinflammation: From Atherosclerosis to COVID-19 | Wagner Dd; Heger La | Atheroschlerosis; COVID-19; Histones; inflammasomes; Neutrophils; Platelets; Protein Arginine Deiminase Type 4 | Arteriosclerosis Thrombosis and Vascular Biology | 10.1161/ATVBAHA.122.317162 |
| 6 | Thromboinflammation in COVID-19 Acute Lung injury | Mitchell Wb | COVID-19; Acute Lung injury; Thrombosis; Inflammation | Paediatric Respiratory Reviews | 10.1016/j.prrv.2020.06.004 |
| 7 | C-Reactive Protein, Immunothrombosis and Venous Thromboembolism | Dix C; Zeller J; Stevens H; Eisenhardt Su; Shing Ksct; Nero Tl; Morton Cj; Parker Mw; Peter K; Mcfadyen Jd | C-Reactive Protein; Venous Thromboembolism; Immunothrombosis; COVID-19; Thromboinflammation | Frontiers in Immunology | 10.3389/fimmu.2022.1002652 |
| 8 | CLEC5A and TLR2 are Critical in SARS-Cov-2-Induced Net Formation and Lung Inflammation | Sung Ps; Yang Sp; Peng Yc; Sun Cp; Tao Mh; Hsieh Sl | Platelets; COVID-19; Neutrophil Extracellular Traps; CLEC2; CLEC5A; TLR2; Immunothrombosis; SARS-Cov-2; Acute Respiratory Distress Syndrome; Spike Protein | Journal of Biomedical Science | 10.1186/s12929-022-00832-z |
| 9 | Fibrinolysis in COVID-19: Impact on Clot Lysis and Modulation of Inflammation | Sugimoto Ma; Perucci Lo; Tavares Lp; Teixeira Mm; Sousa Lp | COVID-19; Fibrinolysis; Plasminogen; Plasmin; Inflammation; Inflammation Resolution; Lung injury; ARDS; Fibrinolytic Drugs; Thromboinflammation; Immunothrombosis | Current Drug Targets | 10.2174/1389450123666221011102250 |
| 10 | Antithrombin Activity is Associated with Persistent Thromboinflammation and Mortality in Patients with Severe COVID-19 Illness | Chen-Goodspeed A; Dronavalli G; Zhang X; Podbielski Jm; Patel B; Modis K; Cotton Ba; Wade Ce; Cardenas Jc | Antithrombin; COVID-19; Hypercoagulability; SARS-Cov-2; Thromboinflammation | Acta Haematologica | 10.1159/000528584 |
| 11 | Complement Contributions to COVID-19 | Conway Em; Pryzdial Elg | Complement System; COVID-19; Endothelium; Innate Immunity; Kallikrein-Kinin; Neutrophil Extracellular Traps; Neutrophils; Platelets; SARS-Cov-2 | Current Opinion in Hematology | 10.1097/MOH.0000000000000724 |
| 12 | Binding of Phosphatidylserine-Positive Microparticles by PBMC Classifies Disease Severity in COVID-19 Patients | Rausch L; Lutz K; Schifferer M; Winheim E; Gruber R; Oesterhaus Ef; Rinke L; Hellmuth Jc; Scherer C; Muenchhoff M; Mandel C; Bergwelt-Baildon M; Simons M; Straub T; Krug Ab; Kranich J; Brocker T | Apoptosis; Cd8^+^ T Cells; COVID-19; Lymphopenia; Phosphatidylserine; Platelet-Derived Microparticle; SARS-Cov-2; Thromboinflammation | Journal of Extracellular Vesicles | 10.1002/jev2.12173 |
| 13 | Platelet Dysregulation in the Pathobiology of COVID-19 | Mellema Ra; Crandell J; Petrey Ac | Platelets; COVID-19; SARS-Cov-2; Immunothrombosis | Hamostaseologie | 10.1055/a-1646-3392 |
| 14 | The Role of Platelets, Neutrophils and Endothelium in COVID-19 Infection | Falcinelli E; Petito E; Gresele P | Antithrombotic Therapy; COVID-19; Cytokines; Endothelial Cells; Extracellular Microvescicles; Neutrophils; Platelets; SARS-Cov-2; Thromboinflammation | Expert Review of Hematology | 10.1080/17474086.2022.2110061 |
| 15 | Thromboinflammation: Dynamics of Physiological and Pathological Interactions Between Inflammation and Coagulation | Stefanski Al; Nitschke E; Dorner T | Inflammation; Coagulation; Thromboinflammation; Autoimmunity; COVID-19 | Aktuelle Rheumatologie | 10.1055/a-1947-5200 |
| 16 | Modulation of Thromboinflammation in Hospitalized COVID-19 Patients with Aprotinin, Low Molecular Weight Heparin, and Anakinra: The Dawn-Antico Study | Engelen Mm; Van Thillo Q; Betrains A; Gyselinck I; Martens Cp; Spalart V; Ockerman A; Devooght C; Wauters J; Gunst J; Wouters C; Vandenbriele C; Rex S; Liesenborghs L; Wilmer A; Meersseman P; Van Den Berghe G; Dauwe D; Belmans A; Thomeer M; Fivez T; Mesotten D; Ruttens D; Heytens L; Dapper I; Tuyls S; De Tavernier B; Verhamme P; Vanassche T | Anakinra; Aprotinin; COVID-19; Heparin; Inflammation; Low-Molecular-Weight; Thrombosis | Research and Practice in Thrombosis and Haemostasis | 10.1002/rth2.12826 |
| 17 | The Role of Glutathione in Prevention of COVID-19 Immunothrombosis: A Review | Glassman I; Le N; Mirhosseini M; Alcantara Ca; Asif A; Goulding A; Muneer S; Singh M; Robison J; Guilford F; Venketaraman V | Glutathione; GSH; COVID-19; SARS-Cov-2; Immunothrombosis; HIV; Diabetes; Microclot; Thrombosis | Frontiers in Bioscience-Landmark | 10.31083/j.fbl2803059 |
| 18 | From Angiotensin-Converting Enzyme 2 Disruption to Thromboinflammatory Microvascular Disease: A Paradigm Drawn from COVID-19 | Vinci R; Pedicino D; andreotti F; Russo G; D'Aiello A; De Cristofaro R; Crea F; Liuzzo G | 2019 Coronavirus; Renin-Angiotensin System; Thromboinflammatoty Microangiopathy; Complement; Von Willebrand Factor | International Journal of Cardiology | 10.1016/j.ijcard.2020.11.016 |
| 19 | The Emerging Role of Neutrophils in the Pathogenesis of Thrombosis in COVID-19 | Iliadi V; Konstantinidou I; Aftzoglou K; Iliadis S; Konstantinidis Tg; Tsigalou C | COVID-19; SARS Cov-2; NETs; Immunothrombosis | International Journal of Molecular Sciences | 10.3390/ijms22105368 |
| 20 | Incidence of Deep Venous Thrombosis in Patients with COVID-19 and Pulmonary Embolism Compression Ultrasound COVID Study | Franco-Moreno A; Herrera-Morueco M; Mestre-Gomez B; Munoz-Rivas N; Abad-Motos A; Salazar-Chiriboga D; Duffort-Falco M; Medrano-Izquierdo P; Bustamante-Fermosel A; Pardo-Guimera V; Ulla-Anes M; Torres-Macho J | Compression Ultrasound; Coronavirus Disease 2019; COVID-19; Deep Venous Thrombosis; Pulmonary Embolism; Thromboinflammatory Syndrome | Journal of Ultrasound in Medicine | 10.1002/jum.15524 |
| 21 | Association of SARS-Cov-2 Nucleocapsid Viral Antigen and the Receptor for Advanced Glycation End Products with Development of Severe Disease in Patients Presenting to the Emergency Department with COVID-19 | Matthay Za; Fields At; Wick Kd; Jones C; Lane Hc; Herrera K; Nunez-Garcia B; Gennatas E; Hendrickson Cm; Kornblith Ae; Matthay Ma; Kornblith Lz | COVID-19; Biomarkers; Thromboinflammation; Receptor for Advanced Glycation End Products; Triage | Frontiers in Immunology | 10.3389/fimmu.2023.1130821 |
| 22 | C-C Motive Chemokine Ligand 2 and Thromboinflammation in COVID-19-Associated Pneumonia: A Retrospective Study | Nieri D; Neri T; Barbieri G; Moneta S; Morelli G; Mingardi D; Spinelli S; Ghiadoni L; Falcone M; Tiseo G; Menichetti F; Franzini M; Caponi L; Paolicchi A; Pancani R; Pistelli F; Carrozzi L; Celi A | CCL2; Thrombosis; Inflammation; COVID-19; Acute Lung injury | Thrombosis Research | 10.1016/j.thromres.2021.06.003 |
| 23 | Extracellular Vesicles: New Players in the Mechanisms of Sepsis-and COVID-19-Related Thromboinflammation | Schiavello M; Vizio B; Bosco O; Pivetta E; Mariano F; Montrucchio G; Lupia E | Extracellular Vesicles; Sepsis; COVID-19; Thromboinflammation | International Journal of Molecular Sciences | 10.3390/ijms24031920 |
| 24 | Vascular Neutrophilic Inflammation and Immunothrombosis Distinguish Severe COVID-19 from influenza Pneumonia | Nicolai L; Leunig A; Brambs S; Kaiser R; Joppich M; Hoffknecht Ml; Gold C; Engel A; Polewka V; Muenchhoff M; Hellmuth Jc; Ruhle A; Ledderose S; Weinberger T; Schulz H; Scherer C; Rudelius M; Zoller M; Keppler Ot; Zwissler B; Von Bergwelt-Baildon M; Kaab S; Zimmer R; Bulow Rd; Von Stillfried S; Boor P; Massberg S; Pekayvaz K; Stark K | COVID-19; Immunopathology; Immunothrombosis; Monocytes; Neutrophils; SARS-Cov-2 | Journal of Thrombosis and Haemostasis | 10.1111/jth.15179 |
| 25 | COVID-19, Immunothrombosis and Venous Thromboembolism: Biological Mechanisms | Loo J; Spittle Da; Newnham M | Pulmonary Embolism; Innate Immunity; Viral infection; Cytokine Biology; Respiratory infection | Thorax | 10.1136/thoraxjnl-2020-216243 |
| 26 | Complement C3 Vs C5 Inhibition in Severe COVID-19: Early Clinical Findings Reveal Differential Biological Efficacy | Mastellos Dc; Da Silva Bgpp; Fonseca Bal; Fonseca Np; Auxiliadora-Martins M; Mastaglio S; Ruggeri A; Sironi M; Radermacher P; Chrysanthopoulou A; Skendros P; Ritis K; Manfra I; Iacobelli S; Huber-Lang M; Nilsson B; Yancopoulou D; Connolly Es; Garlanda C; Ciceri F; Risitano Am; Calado Rt; Lambris Jd | COVID-19; Thromboinflammation; C3 inhibition; C5 Blockade; AMY-101; Eculizumab; Drug Efficacy; Biomarkers | Clinical Immunology | 10.1016/j.clim.2020.108598 |
| 27 | COVID-19 and Immunothrombosis: Emerging Understanding and Clinical Management | Shaw Rj; Bradbury C; Abrams St; Wang Gz; Toh Ch | COVID-19; Immunothrombosis; Coagulopathy; Anticoagulation; Immunomodulatory | British Journal of Haematology | 10.1111/bjh.17664 |
| 28 | Mechanisms of Immunothrombosis by SARS-Cov-2 | Hernandez-Huerta Mt; Perez-Santiago Ad; Mayoral Lpc; Navarro Lms; Canales Fjr; Majluf-Cruz A; Matias-Cervantes Ca; Mayoral Epc; Diaz Cr; Mayoral-andrade G; Cruz Mm; Angel Jl; Perez-Campos E | Pathogen-Associated Molecular Patterns; Damage-Associated Molecular Patterns; Extracellular DNA; Extracellular RNA; SARS-Cov-2; Immunothrombosis | Biomolecules | 10.3390/biom11111550 |
| 29 | Activated Platelets and Platelet-Derived Extracellular Vesicles Mediate COVID-19-Associated Immunothrombosis | Ebeyer-Masotta M; Eichhorn T; Weiss R; Laukova L; Weber V | Apheresis; Coagulopathy; COVID-19; Extracellular Vesicles; Heparin; Immunothrombosis; Platelets; Platelet Factor 4 | Frontiers in Cell and Developmental Biology | 10.3389/fcell.2022.914891 |
| 30 | Phosphatidylserine is an Overlooked Mediator of COVID-19 Thromboinflammation | Lind Se | Coagulation; Thrombosis; Phosphatidylserine; Thromboinflammation; COVID-19 | Heliyon | 10.1016/j.heliyon.2021.e06033 |
| 31 | Immunothrombosis Biomarkers as Potential Predictive Factors of Acute Respiratory Distress Syndrome in Moderate-To-Critical COVID-19: A Single-Center, Retrospective Cohort Study | Yiang Gt; Wu Yk; Tsai Kw; Tzeng Is; Hu Wc; Liao Mt; Lu Kc; Chung Hw; Chao Yc; Su Wl | ARDS; COVID-19; Histone H3; Human Factor XII; Immunothrombosis; Tissue Factor; Von Willebrand Factor | Immunology Letters | 10.1016/j.imlet.2023.01.010 |
| 32 | COVID-19 and Immunothrombosis: Pathophysiology and Therapeutic Implications | Lim Ms; Mcrae S | COVID-19; Coagulopathy; Immunothrombosis; Pathophysiology; Venous Thromboembolism | Critical Reviews in Oncology Hematology | 10.1016/j.critrevonc.2021.103529 |
| 33 | Abnormal Immunothrombosis and Lupus Anticoagulant in a Catastrophic COVID-19 Recalling Asherson's Syndrome | Roncati L; Corsi L; Barbolini G | Coronavirus Disease 2019 (COVID-19); Abnormal Immunothrombosis; Lupus Anticoagulant (LAC); Megakaryocytes; Antiphospholipid Syndrome (Hughes Syndrome); Asherson’s Syndrome | Journal of Thrombosis and Thrombolysis | 10.1007/s11239-021-02444-0 |
| 34 | Platelets: 'Multiple Choice' Effectors in the Immune Response and their Implication in COVID-19 Thromboinflammatory Process | Rolla R; Puricelli C; Bertoni A; Boggio E; Gigliotti Cl; Chiocchetti A; Cappellano G; Dianzani U | COVID-19; Immune Response Modulator; Infection; Platelet-Derived Extracellular Vesicles; Thromboinflammation | International Journal of Laboratory Hematology | 10.1111/ijlh.13516 |
| 35 | Thrombopoietin Participates in Platelet Activation in COVID-19 Patients | Lupia E; Capuano M; Vizio B; Schiavello M; Bosco O; Gelardi M; Favale E; Pivetta E; Morello F; Husain S; Keshavjee S; Del Sorbo L; Montrucchio G | COVID-19; Thrombopoietin; Interleukin-6; Platelet Activation; Biomarker; Thromboinflammation | Ebiomedicine | 10.1016/j.ebiom.2022.104305 |
| 36 | Endothelial Dysfunction and Immunothrombosis as key Pathogenic Mechanisms in COVID-19 | Bonaventura A; Vecchie A; Dagna L; Martinod K; Dixon Dl; Van Tassell Bw; Dentali F; Montecucco F; Massberg S; Levi M; Abbate A | NA | Nature Reviews Immunology | 10.1038/s41577-021-00536-9 |
| 37 | Preventing the Development of Severe COVID-19 By Modifying Immunothrombosis | Morris G; Bortolasci Cc; Puri Bk; Olive L; Marx W; O'Neil A; Athan E; Carvalho A; Maes M; Walder K; Berk M | COVID-19; SARS-Cov-2; Respiratory Infection; Treatment | Life Sciences | 10.1016/j.lfs.2020.118617 |
| 38 | Platelet-Leukocyte Interactions in COVID-19: Contributions to Hypercoagulability, Inflammation, and Disease Severity | Hottz Ed; Bozza Pt | COVID-19; Monocytes; Neutrophils; Platelets; Thromboinflammation | Research and Practice in Thrombosis and Haemostasis | 10.1002/rth2.12709 |
| 39 | "War to the Knife" Against Thromboinflammation to Protect Endothelial Function of COVID-19 Patients | Guglielmetti G; Quaglia M; Sainaghi Pp; Castello Lm; Vaschetto R; Pirisi M; Della Corte F; Avanzi Gc; Stratta P; Cantaluppi V | COVID-19; Thrombosis; Thromboinflammation; Heparin; Plasma; Endothelial Dysfunction; Complement; Extracellular Vesicles; Anticoagulation | Critical Care | 10.1186/s13054-020-03060-9 |
| 40 | Mechanisms of COVID-19 Associated Pulmonary Thrombosis: A Narrative Review | Niculae Cm; Hristea A; Moroti R | COVID-19; SARS-Cov-2 Infection; Pulmonary in Situ Thrombosis; Embolism; Immunothrombosis; Inflammation; Coagulopathy | Biomedicines | 10.3390/biomedicines11030929 |
| 41 | COVID-19-Associated Coagulopathy: An Exacerbated Immunothrombosis Response | Jayarangaiah A; Kariyanna Pt; Chen Xy; Jayarangaiah A; Kumar A | COVID-19; Immunothrombosis; Coagulopathy | Clinical and Applied Thrombosis-Hemostasis | 10.1177/1076029620943293 |
| 42 | Thromboinflammation Supports Complement Activation in Cancer Patients with COVID-19 | Peerschke Ei; Valentino A; So Rj; Shulman S; Ravinder | COVID-19; Cancer; Complement; Thromboinflammation; Endothelial Dysfunction | Frontiers in Immunology | 10.3389/fimmu.2021.716361 |
| 43 | Mechanisms of Immunothrombosis in COVID-19 | Portier I; Campbell Ra; Denorme F | Coagulopathy; Coronavirus Disease 2019; Immunothrombosis; Neutrophil Extracellular Traps; Platelets | Current Opinion in Hematology | 10.1097/MOH.0000000000000666 |
| 44 | Autoantibodies Stabilize Neutrophil Extracellular Traps in COVID-19 | Zuo Y; Yalavarthi S; Navaz S; Hoy C; Harbaugh A; Gockman K; Zuo M; Madison Ja; Shi H; Kanthi Y; Knight Js | Adaptive Immunity; Autoimmunity; COVID-19; Neutrophils | JCI Insight | 10.1172/jci.insight.150111 |
| 45 | Transcriptional Landscape of Circulating Platelets from Patients with COVID-19 Reveals Key Subnetworks and Regulators Underlying SARS-Cov-2 Infection: Implications for Immunothrombosis | Ji Wp; Chen L; Yang W; Li K; Zhao Jt; Yan Cc; You Cc; Jiang Mh; Zhou M; Shen X | COVID-19; SARS-Cov-2; Platelets; Immunothrombosis; Transcriptome | Cell and Bioscience | 10.1186/s13578-022-00750-5 |
| 46 | Infection with SARS-Cov-2 is Associated with Elevated Levels of IP-10, MCP-1, and IL-13 in Sepsis Patients | Eichhorn T; Huber S; Weiss R; Ebeyer-Masotta M; Laukova L; Emprechtinger R; Bellmann-Weiler R; Lorenz I; Martini J; Pirklbauer M; Orth-Hoeller D; Wuerzner R; Weber V | Sepsis; COVID-19; Immunothrombosis; Extracellular Vesicles; Inflammation | Diagnostics | 10.3390/diagnostics13061069 |
| 47 | COVID-19: Lung-Centric Immunothrombosis | Kvietys Pr; Fakhoury Hma; Kadan S; Yaqinuddin A; Al-Mutairy E; Al-Kattan K | Acute Respiratory Distress Syndrome (ARDS); Coronavirus; COVID-19; Cytokine Storm; NET; SARS-Cov-2 | Frontiers in Cellular and Infection Microbiology | 10.3389/fcimb.2021.679878 |
| 48 | A Randomized, Open-Label, Adaptive, Proof-of-Concept Clinical Trial of Modulation of Host Thromboinflammatory Response in Patients with COVID-19: The Dawn-Antico Study | Vanassche T; Engelen Mm; Van Thillo Q; Wauters J; Gunst J; Wouters C; Vandenbriele C; Rex S; Liesenborghs L; Wilmer A; Meersseman P; Van Den Berghe G; Dauwe D; Verbeke G; Thomeer M; Fivez T; Mesotten D; Ruttens D; Heytens L; Dapper I; Tuyls S; De Tavernier B; Verhamme P | COVID-19; SARS-Cov-2; Low Molecular Weight Heparins; Aprotinin; Anakinra; Thromboinflammatory Response; Thrombosis; Inflammation | Trials | 10.1186/s13063-020-04878-y |
| 49 | Effects of The Circulating Environment of COVID-19 on Platelet and Neutrophil Behavior | Fields At; Andraska Ea; Kaltenmeier C; Matthay Za; Herrera K; Nunez-Garcia B; Jones Cm; Wick Kd; Liu Sl; Luo Jh; Yu Yp; Matthay Ma; Hendrickson Cm; Bainton Rj; Barrett Tj; Berger Js; Neal Md; Kornblith Lz | Blood Platelets; Neutrophil Extracellular Traps; COVID-19; Thromboinflammation; Plasma | Frontiers in Immunology | 10.3389/fimmu.2023.1130288 |
| 50 | A Proof of Evidence Supporting Abnormal Immunothrombosis in Severe COVID-19: Naked Megakaryocyte Nuclei Increase in the Bone Marrow and Lungs of Critically Ill Patients | Roncati L; Ligabue G; Nasillo V; Lusenti B; Gennari W; Fabbiani L; Malagoli C; Gallo G; Giovanella S; Lupi M; Salviato T; Paolini A; Costantini M; Trenti T; Maiorana A | Coronavirus Disease 2019 (COVID-19); Immunothrombosis; Interleukin-6 (IL-6); Megakaryocytes; Naked Megakaryocyte Nuclei; Severe Acute Respiratory Syndrome Coronavirus 2 (SARS-Cov-2) | Platelets | 10.1080/09537104.2020.1810224 |
| 51 | Kidney in The NET of Acute and Long-Haul Coronavirus Disease 2019: A Potential Role for Lipid Mediators in Causing Renal injury and Fibrosis | Chiang Kc; Imig Jd; Kalantar-Zadeh K; Gupta A | Acute Kidney Injury; Coronavirus Disease 2019; Prostaglandin D-2; Thromboinflammation; Thromboxane | Current Opinion in Nephrology and Hypertension | 10.1097/MNH.0000000000000750 |
| 52 | Clinical Applications of Thrombopoietin Silencing: A Possible Therapeutic Role in COVID-19? | Alentado Vj; Moliterno Ar; Srour Ef; Kacena Ma | Coronavirus 2019; COVID-19; Thromboinflammation; Thrombopoietin; Thrombopoietin Silencing; Megakaryocytes | Cytokine | 10.1016/j.cyto.2021.155634 |
| 53 | NETosis and Neutrophil Extracellular Traps in COVID-19: Immunothrombosis and Beyond | Zhu Yf; Chen Xl; Liu X | NETosis; NETs; COVID-19; Immunothrombosis; Post COVID-19 Syndrome; Immunopathology | Frontiers in Immunology | 10.3389/fimmu.2022.838011 |
| 54 | Pulmonary Thromboembolic Events in COVID-19-A Systematic Literature Review | Overton Pm; Toshner M; Mulligan C; Vora P; Nikkho S; De Backer J; Lavon B; Klok Fa | Incidence; Inflammation; Pulmonary Embolism; Pulmonary Hypertension; SARS-Cov-2 | Pulmonary Circulation | 10.1002/pul2.12113 |
| 55 | Role of CD39 in COVID-19 Severity: Dysregulation of Purinergic Signaling and Thromboinflammation | Diaz-Garcia E; Garcia-Tovar S; Alfaro E; Zamarron E; Mangas A; Galera R; Ruiz-Hernandez Jj; Sole-Violan J; Rodriguez-Gallego C; Van-Den-Rym A; Perez-De-Diego R; Nanwani-Nanwani K; Lopez-Collazo E; Garcia-Rio F; Cubillos-Zapata C | COVID-19; Thromboinflammation; CD39; Purinergic Dysregulation; Hypoxia | Frontiers in Immunology | 10.3389/fimmu.2022.847894 |
| 56 | Mechanisms of Immunothrombosis in Vaccine-induced Thrombotic Thrombocytopenia (VITT) Compared to Natural SARS-Cov-2 Infection | Mcgonagle D; De Marco G; Bridgewood C | COVID-19 Pneumonia Related Thrombosis; Heparin Induced Thrombocytopenia (HIT); DNA-PF4 Interactions; VITT Model; Vaccine Induced Thrombotic Thrombocytopenia (VITT) | Journal of Autoimmunity | 10.1016/j.jaut.2021.102662 |
| 57 | The Coagulopathy, Endotheliopathy, and Vasculitis of COVID-19 | Iba T; Connors Jm; Levy Jh | COVID-19; Thromboembolism; Coagulopathy; Endotheliopathy; Vasculitis | Inflammation Research | 10.1007/s00011-020-01401-6 |
| 58 | Immunothrombotic Dysregulation in Chagas Disease and COVID-19: A Comparative Study of Anticoagulation | Mayoral Lpc; Hernandez-Huerta Mt; Papy-Garca D; Barritault D; Zenteno E; Navarro Lms; Mayoral Epc; Cervantes Cam; Cruz Mm; Andrade Gm; Cervantes Ml; Martnez Gv; Sanchez Cl; Canseco Sp; Cruz Rm; Prez-Campos E | COVID-19; Hypercoagulability; Platelet; Hyperaggregability; Immunothrombosis; SARS-Cov-2 | Molecular and Cellular Biochemistry | 10.1007/s11010-021-04204-3 |
| 59 | Microvascular Lung Vessels Obstructive Thromboinflammatory Syndrome in Patients with COVID-19: Insights from Lung Intravascular Optical Coherence Tomography | Hajjar La; Ancona Mb; Kalil R; Tresoldi M; Caldas Jg; Monti G; Carnevale Fc; De Cobelli F; De Assis Am; Ciceri F; Landoni G; Dijkstra J; Moroni F; Abizaid Aac; Ungaretti Fw; Carmona Mjc; De Backer D; Pompilio Ce; Jr Fsd; Campos Cm; Zangrillo A; Montorfano M | COVID-19; Microclots; OCT; Thrombo-Inflammatory Syndrome; D-Dimer | Frontiers in Medicine | 10.3389/fmed.2023.1050531 |
| 60 | Neutrophil Extracellular Traps Contribute to Immunothrombosis in COVID-19 Acute Respiratory Distress Syndrome | Middleton Ea; He Xy; Denorme F; Campbell Ra; Ng D; Salvatore Sp; Mostyka M; Baxter-Stoltzfus A; Borczuk Ac; Loda M; Cody Mj; Manne Bk; Portier I; Harris Es; Petrey Ac; Beswick Ej; Caulin Af; Iovino A; Abegglen Lm; Weyrich As; Rondina Mt; Egeblad M; Schiffman Jd; Yost Cc | COVID-19, Respiratory Distress Syndrome, Adult, Blood Platelets, Plasma, Neutrophils, Immunothrombosis, Neutrophil Extracellular Traps | Blood | 10.1182/blood.2020007008 |
| 61 | COVID-19 Coagulopathy-What Should We Treat? | Chowdary P | Anticoagulants; ARDS; Coagulation; Coronavirus; COVID-19; Covid Coagulopathy; Fibrinolytics; Hypercoagulability; Pulmonary Microvascular Thrombosis; Thromboinflammation | Experimental Physiology | 10.1113/EP089404 |
| 62 | Targeting Thromboinflammation in COVID-19-A Narrative Review of the Potential of C1 Inhibitor to Prevent Disease Progression | Urwyler P; Moser S; Trendelenburg M; Sendi P; Osthoff M | COVID-19; C1 Inhibitor; Complement System; Thromboinflammation; Contact Activation System; Kallikrein-Kinin System | Molecular Immunology | 10.1016/j.molimm.2022.08.008 |
| 63 | The Outcome of Critically Ill COVID-19 Patients is Linked to Thromboinflammation Dominated by the Kallikrein/Kinin System | Lipcsey M; Persson B; Eriksson O; Blom Am; Fromell K; Hultstrom M; Huber-Lang M; Ekdahl Kn; Frithiof R; Nilsson B | Thromboinflammation; Kallikrein; Kinin System; Complement System; Coagulation System; Fibrinolysis System; COVID-19; Prognosis | Frontiers in Immunology | 10.3389/fimmu.2021.627579 |
| 64 | Antithrombotic Therapy in Patients with COVID-19? -Rationale and Evidence- | Godino C; Scotti A; Maugeri N; Mancini N; Fominskiy E; Margonato A; Landoni G | Anticoagulants; Antiplatelet; Antithrombotic Therapy; Coronavirus; COVID-19; Thromboinflammatory Syndrome | International Journal of Cardiology | 10.1016/j.ijcard.2020.09.064 |
| 65 | Carotid Free-Floating Thrombus in COVID-19: A Cerebrovascular Disorder of Cytokine Storm-Related Immunothrombosis | Pensato U; Forlivesi S; Gentile M; Romoli M; Muccioli L; Ambrosi F; Foschini Mp; Gallo C; Ballestrazzi Ms; Teutonico P; Faggioli G; Gargiulo M; Galluzzo S; Taglialatela F; Simonetti L;Zini A | Ischemic Stroke; Treatment; Atherosclerosis; Inflammation; SARS-Cov-2 | Neurological Sciences | 10.1007/s10072-023-06682-3 |
| 66 | Thromboinflammation in Coronavirus Disease 2019: The Clot Thickens | Iffah R; Gavins Fne | Inflammation; Neutrophils; Platelets; Resolution Pharmacology; Thromboinflammation; Thrombosis | British Journal of Pharmacology | 10.1111/bph.15594 |
| 67 | Inflammation and Thrombosis in COVID-19 Pathophysiology: Proteinase-Activated and Purinergic Receptors ss Drivers and Candidate Therapeutic Targets | Sriram K; Insel Pa | Angiotensin; Endothelium; GPCR; Platelets; Thrombin | Physiological Reviews | 10.1152/physrev.00035.2020 |
| 68 | Endothelial Thrombomodulin Downregulation Caused by Hypoxia Contributes to Severe Infiltration and Coagulopathy in COVID-19 Patient Lungs | Won T; Wood Mk; Hughes Dm; Talor Mv; Ma Zx; Schneider J; Skinner Jt; Asady B; Goerlich E; Halushka Mk; Hays Ag; Kim Dh; Parikh Cr; Rosenberg Az; Coppens I; Johns Ra; Gilotra Na; Hooper Je; Pekosz A; Cihakova D | COVID-19; SARS-Cov-2; Immunothrombosis; Endothelial Cell Dysfunction; Thrombomodulin | Ebiomedicine | 10.1016/j.ebiom.2022.103812 |
| 69 | Pathomechanisms Underlying Hypoxemia in Two COVID-19-Associated Acute Respiratory Distress Syndrome Phenotypes: Insights from Thrombosis and Hemostasis | Gando S; Wada T | Acute Respiratory Distress Syndrome (ARDS); Compliance; COVID-19; Hypoxemia; Neutrophil Extracellular Traps (NETs); Shunt; Thrombosis | Shock | 10.1097/SHK.0000000000001825 |
| 70 | Thromboinflammation Response to Tocilizumab in COVID-19 | Gergi M; Cushman M; Littenberg B; Budd Rc | COVID-19; COVID-19 Coagulopathy; Interleukin-6; Thromboinflammation; Tocilizumab | Research and Practice in Thrombosis and Haemostasis | 10.1002/rth2.12436 |
| 71 | NETosis and SARS-Cov-2 infection Related Thrombosis: A Narrative Review | Behzadifard M; Soleimani M | SARS-Cov-2; COVID-19; Thrombosis; Tissue Factor; Angiotensin Converting Enzyme; NETosis; NETs; Factor Seven Activating Protease (FSAP); Tissue Factor Pathway inhibitor; TFPI; Acute Respiratory Distress Syndrome; ARDS | Thrombosis Journal | 10.1186/s12959-022-00375-1 |
| 72 | Thromboinflammation in COVID-19: Can Alpha (2)-Macroglobulin Help to Control the Fire? | Seitz R; Gurtler L; Schramm W | COVID-19; Alpha (2)-Macroglobulin; Endothelial Cells; Extracellular Traps; Neutrophil; Thromboinflammation | Journal of Thrombosis and Haemostasis | 10.1111/jth.15190 |
| 73 | COVID-19 and Antiphospholipid Antibodies | Butt A; Erkan D; Lee Ai | COVID-19 Coagulopathy; Antiphospholipid Syndrome; Antiphospholipid Antibodies; Lupus Anticoagulant; Anticardiolipin Antibodies; Beta-2 Glycoprotein-1 Antibodies; COVID-19 Vaccination; Immunothrombosis | Best Practice & Research Clinical Haematology | 10.1016/j.beha.2022.101402 |
| 74 | Immunothrombosis in Acute Respiratory Dysfunction of COVID-19 | Fang Xz; Wang Yx; Xu Ja; He Yj; Peng Zk; Shang Y | COVID-19; Inflammation; Thrombosis; Respiratory Dysfunction; Therapeutic | Frontiers in Immunology | 10.3389/fimmu.2021.651545 |
| 75 | Venous Thromboembolic Disease in COVID-19, Pathophysiology, Therapy and Prophylaxis | Dybowska M; Wyrostkiewicz D; Opoka L; Lewandowska K; Sobiecka M; Tomkowski W; Szturmowicz M | COVID-19; SARS-Cov-2; Venous Thromboembolism; NETs; Immunothrombosis; Thromboprophylaxis; Anticoagulation Treatment | International Journal of Molecular Sciences | 10.3390/ijms231810372 |
| 76 | Patients With COVID-19: In the Dark-NETs of Neutrophils | Ackermann M; Anders Hj; Bilyy R; Bowlin Gl; Daniel C; De Lorenzo R; Egeblad M; Henneck T; Hidalgo A; Hoffmann M; Hohberger B; Kanthi Y; Kaplan Mj; Knight Js; Knopf J; Kolaczkowska E; Kubes P; Leppkes M; Mahajan A; Manfredi Aa; Maueroder C; Maugeri N; Mitroulis I; Munoz Le; Narasaraju T; Naschberger E; Neeli I; Ng Lg; Radic Mz; Ritis K; Rovere-Querini P; Schapher M; Schauer C;Simon Hu; Singh J; Skendros P; Stark K;Sturzl M; Van Der Vlag J; Vandenabeele P; Vitkov L; Von Kockritz-Blickwede M; Yanginlar C; Yousefi S; Zarbock A; Schett G; Herrmann M | NA | Cell Death and Differentiation | 10.1038/s41418-021-00805-z |
| 77 | Complement and Tissue Factor-Enriched Neutrophil Extracellular Traps Are Key Drivers in COVID-19 Immunothrombosis | Skendros P; Mitsios A; Chrysanthopoulou A; Mastellos Dc; Metallidis S; Rafailidis P; Ntinopoulou M; Sertaridou E; Tsironidou V; Tsigalou C; Tektonidou M; Konstantinidis T; Papagoras C; Mitroulis I; Germanidis G; Lambris Jd; Ritis K | COVID-19; Complement; Immunology; Neutrophils; Thrombosis | Journal of Clinical Investigation | 10.1172/JCI141374 |
| 78 | Anticoagulation in Coronavirus Disease 2019 (COVID-19): Confirmed and Controversial Aspects | Rauch-Krohnert U; Riess H | COVID-19; Prognosis; Venous Thrombosis; Microthrombosis; Heparin; Fondaparinux | Internist | 10.1007/s00108-022-01296-x |
| 79 | Immunothrombotic Dysregulation in COVID-19 Pneumonia is Associated with Respiratory Failure and Coagulopathy | Nicolai L; Leunig A; Brambs S; Kaiser R; Weinberger T; Weigand M; Muenchhoff M; Hellmuth Jc; Ledderose S; Schulz H; Scherer C; Rudelius M; Zoller M; Hochter D; Keppler O; Teupser D; Zwissler B; Von Bergwelt-Baildon M; Kaab S; Massberg S; Pekayvaz K; Stark K | Blood Platelets; COVID-19; Disseminated Intravascular Coagulation; Neutrophils; Respiratory Insufficiency; Severe Acute Respiratory Syndrome Coronavirus 2; Thrombosis | Circulation | 10.1161/CIRCULATIONAHA.120.048488 |
| 80 | Neutrophil Activation and Neutrophil Extracellular Traps (NETs) in COVID-19 ARDS and Immunothrombosis | Cesta Mc; Zippoli M; Marsiglia C; Gavioli Em; Cremonesi G; Khan A; Mantelli F; Allegretti M; Balk R | ARDS; COVID-19; CXCL8; Immunomodulators | European Journal of Immunology | 10.1002/eji.202250010 |
| 81 | Lung-Centric Inflammation of COVID-19: Potential Modulation by Vitamin D | Fakhoury Hma; Kvietys Pr; Shakir I; Shams H; Grant Wb; Alkattan K | Acute Respiratory Distress Syndrome (ARDS); Coronavirus; COVID-19; Cytokine Storm; Inflammasome; Neutrophil Extracellular Traps (NETs); SARS-Cov-2; Vitamin D | Nutrients | 10.3390/nu13072216 |
| 82 | Therapeutic Implications of Ongoing Alveolar Viral Replication in COVID-19 | Mcgonagle D; Kearney Mf; O'Regan A; O'Donnell Js; Quartuccio L; Watad A; Bridgewood C | NA | Lancet Rheumatology | 10.1016/S2665-9913(21)00322-2 |
| 83 | Elevated MYL9 Reflects the MYL9-Containing Microthrombi in SARS-Cov-2-Induced Lung Exudative Vasculitis and Predicts COVID-19 Severity | Iwamura C; Hirahara K; Kiuchi M; Ikehara S; Azuma K; Shimada T; Kuriyama S; Ohki S; Yamamoto E; Inaba Y; Shiko Y; Aoki A; Kokubo K; Hirasawa R; Hishiya T; Tsuji K; Nagaoka T; Ishkawa S; Kojima A; Mito H; Hase R; Kasahara Y; Kuriyama N; Tsukamoto T; Nakamura S; Urushibara T; Kaneda S; Sakao S; Tobiume M; Suzuki Y; Tsujiwaki M; Kubo T; Hasegawa T; Nakase H; Nishida O; Takahashi K; Baba K; Iizumi Y; Okazaki T; Kimura My; Yoshino I; Igari H; Nakajima H; Suzuki T; Hanaoka H; Nakada T; Ikehara Y; Yokote K; Nakayama T | COVID-19; Exudative Vasculitis; Nonconventional Monocytes; Microthrombi; Plasma MYL9 | Proceedings of The National Academy of Sciences of the United States of America | 10.1073/pnas.2203437119 |
| 84 | Immune Mechanisms of Pulmonary Intravascular Coagulopathy in COVID-19 Pneumonia | Mcgonagle D; O'Donnell Js; Sharif K; Emery P; Bridgewood C | NA | Lancet Rheumatology | 10.1016/S2665-9913(20)30121-1 |
| 85 | Patients with Autoimmune Chronic Inflammatory Diseases Present Increased Biomarkers of Thromboinflammation and Endothelial Dysfunction in the Absence of Flares and Cardiovascular Comorbidities | Anyfanti P; Gavriilaki E; Nikolaidou B; Yiannaki E; Lazaridis A; Papadopoulos N; Douma S; Doumas M; Gkaliagkousi E | Microvesicles; Autoimmune Rheumatic Diseases; Endothelial Dysfunction; Thromboinflammation | Journal of Thrombosis and Thrombolysis | 10.1007/s11239-021-02517-0 |
| 86 | Pathogenic Basis of Thromboinflammation and Endothelial Injury in COVID-19: Current Findings and Therapeutic Implications | Higashikuni Y; Liu Wh; Obana T; Sata M | COVID-19; Endothelial Injury; Inflammation; Platelet Activation; SARS-Cov-2; Therapeutics; Thrombosis | International Journal of Molecular Sciences | 10.3390/ijms222112081 |
| 87 | Endothelial Cell-Activating Antibodies in COVID-19 | Shi H; Zuo Y; Navaz S; Harbaugh A; Hoy Ck; Gandhi Aa; Sule G; Yalavarthi S; Gockman K; Madison Ja; Wang Jt; Zuo M; Shi Y; Maile Md; Knight Js; Kanthi Y | COVID-19; Autoantibodies; Antiphospholipid Antibodies; Endothelial Cells; Cell Adhesion | Arthritis & Rheumatology | 10.1002/art.42094 |
| 88 | A Critical Review of the Pathophysiology of Thrombotic Complications and Clinical Practice Recommendations for Thromboprophylaxis in Pregnant Patients with COVID-19 | D'Souza R; Malhame I; Teshler L; Acharya G; Hunt Bj; Mclintock C | COVID-19; Pregnancy; SARS-Cov-2; Thromboembolic Complications; Thromboprophylaxis; Venous Thromboembolism | Acta Obstetricia Et Gynecologica Scandinavica | 10.1111/aogs.13962 |
| 89 | Update on Innate Immunity in Acute Kidney Injury-Lessons Taken from COVID-19 | Musial K | Coagulation Cascade; Complement System; Immunothrombosis; Necroinflammation; Neutrophil Extracellular Traps; Tubular Epithelial Cells; Vascular Endothelial Damage | International Journal of Molecular Sciences | 10.3390/ijms232012514 |
| 90 | Immunothrombosis in COVID-19: Implications of Neutrophil Extracellular Traps | Bautista-Becerril B; Campi-Caballero R; Sevilla-Fuentes S; Hernandez-Regino Lm; Hanono A; Flores-Bustamante A; Gonzalez-Flores J; Garcia-Avila Ca; Aquino-Galvez A; Castillejos-Lopez M; Juarez-Cisneros A; Camarena A | Immunothrombosis; COVID-19; Neutrophil Extracellular Traps; CID; SARS-Cov-2 | Biomolecules | 10.3390/biom11050694 |
| 91 | The Role of Neutrophil Extracellular Traps in Acute Lung Injury | Scozzi D; Liao Fy; Krupnick As; Kreisel D; Gelman Ae | NETs (Neutrophil Extracellular Traps); ALI (Acute Lung injury); ARDS (Acute Respiratory Distress Syndrome); Sterile Inflammatory Response; Infections and Sepsis; COVID-19; DAMPs (Damage-Associated Molecular Patterns); Thromboinflammation | Frontiers in Immunology | 10.3389/fimmu.2022.953195 |
| 92 | Coagulopathy and COVID-19 | Lorini Fl; Di Matteo M; Gritti P; Grazioli L; Benigni A; Zacchetti L; Bianchi I; Fabretti F; Longhi L | COVID-19; Coagulopathy; D-Dimer; Immunothrombosis | European Heart Journal Supplements | 10.1093/eurheartj/suab100 |
| 93 | PAD Inhibitors as a Potential Treatment for SARS-Cov-2 Immunothrombosis | Elliott W; Guda Mr; Asuthkar S; Teluguakula N; Prasad Dvr; Tsung Aj; Velpula Kk | SARS-Cov2; COVID-19; PAD; NET | Biomedicines | 10.3390/biomedicines9121867 |
| 94 | Neutrophil Extracellular Traps (NETs) in Severe SARS-Cov-2 Lung Disease | Szturmowicz M; Demkow U | NETs; SARS-Cov-2; COVID-19 Disease; Acute Lung Injury; Immunothrombosis; Cytokine Storm | International Journal of Molecular Sciences | 10.3390/ijms22168854 |
| 95 | Mast Cells in Alveolar Septa of COVID-19 Patients: A Pathogenic Pathway that may link Interstitial Edema to Immunothrombosis | Motta Jd; Miggiolaro Afrd; Nagashima S; De Paula Cbv; Baena Cp; Scharfstein J; De Noronha L | SARS-Cov-2; COVID-19; Mast Cells (MC); Cell-Mediated Immunity; Immune Responses; Interleukin-4 (IL-4) | Frontiers in Immunology | 10.3389/fimmu.2020.574862 |
| 96 | Anticoagulation in Hospitalized Patients with COVID-19 | Kreuziger Lb; Sholzberg M; Cushman M | NA | Blood | 10.1182/blood.2021014527 |
| 97 | Thrombosis and Coagulopathy in COVID-19: An Illustrated Review | Levi M; Hunt Bj | COVID-19; Novel Coronavirus; Thrombosis; Coagulopathy; Coagulation | Research and Practice in Thrombosis and Haemostasis | 10.1002/rth2.12400 |
| 98 | Role of Vitamin K-Dependent Factors Protein S and GAS6 and TAM Receptors in SARS-Cov-2 Infection and COVID-19-Associated Immunothrombosis | Tutusaus A; Mari M; Ortiz-Perez Jt; Nicolaes Gaf; Morales A; De Frutos Pg | AXL; MERTK; GAS6; Viral Infection; Coagulation; Endothelium; Immune Response | Cells | 10.3390/cells9102186 |
| 99 | Von Willebrand Factor Multimer Formation Contributes to Immunothrombosis in Coronavirus Disease 2019 | Doevelaar Aan; Bachmann M; Holzer B; Seibert Fs; Rohn Bj; Bauer F; Witzke O; Dittmer U; Bachmann M; Yilmaz S; Dittmer R; Schneppenheim S; Babel N; Budde U; Westhoff Th | ADAMTS13; Coronavirus Disease 2019; Immunothrombosis; Plasma Exchange; Severe Acute Respiratory Syndrome Coronavirus 2; Von Willebrand Factor | Critical Care Medicine | 10.1097/CCM.0000000000004918 |
| 100 | Activated Neutrophils in the Initiation and Progression of COVID-19: Hyperinflammation and Immunothrombosis in COVID-19 | Zhao Xy; Zhou Lj; Kou Y; Kou Jj | COVID-19; Neutrophil; Cytokine Storm; Neutrophil Extracellular Trap; Immunothrombosis | American Journal of Translational Research | NA (Am J Transl Res 2022;14(3):1454-1468) |
| 101 | Analysis of Transcriptomic Data Sets Supports the Role of IL-6 in NETosis and Immunothrombosis in Severe COVID-19 | Mukhopadhyay S; Sinha S; Mohapatra Sk | COVID-19; IL-6; Neutrophil Extracellular Trap; NETosis; Complement; Coagulation; Transcriptomics | BMC Genomic Data | 10.1186/s12863-021-01001-1 |
| 102 | Complement Activation Predicts Negative Outcomes in COVID-19: The Experience from Northen Italian Patients | Meroni Pl; Croci S; Lonati Pa; Pregnolato F; Spaggiari L; Besutti G; Bonacini M; Ferrigno I; Rossi A; Hetland G; Hollan I; Cugno M; Tedesco F; Borghi Mo; Salvarani C | Complement; Cytokines; COVID-19; Disease Severity; Disease Outcome | Autoimmunity Reviews | 10.1016/j.autrev.2022.103232 |
| 103 | Vascular Damage, Thromboinflammation, Plasmablast Activation, T-Cell Dysregulation and Pathological Histiocytic Response in Pulmonary Draining Lymph Nodes of COVID-19 | Haslbauer Jd; Zinner C; Stalder Ak; Schneeberger J; Menter T; Bassetti S; Mertz Kd; Went P; Matter Ms; Tzankov A | COVID-19; Immunopathology; LYMPH Nodes; Macrophage Activation; Plasmablasts; Thrombosis; Thromboinflammation; T-Cell Dysregulation | Frontiers in Immunology | 10.3389/fimmu.2021.763098 |
| 104 | COVID-19 Vasculitis and Vasculopathy-Distinct Immunopathology Emerging from The Close Juxtaposition of Type II Pneumocytes and Pulmonary Endothelial Cells | Giryes S; Bragazzi Nl; Bridgewood C; De Marco G; Mcgonagle D | SARS-Cov-2; COVID-19 Vaccine; Vasculitis; Vasculopathy; Endotheliitis; Immunothrombosis | Seminars in Immunopathology | 10.1007/s00281-022-00928-6 |
| 105 | Venous Thrombosis and SARS-Cov-2 | Zdanyte M; Rath D; Gawaz M; Geisler T | SARS-Cov-2; COVID-19; Thrombosis; Venous Thromboembolism | Hamostaseologie | 10.1055/a-1661-0283 |
| 106 | Platelet and Extracellular Vesicles in COVID-19 Infection and its Vaccines | Goubran H; Seghatchian J; Sabry W; Ragab G; Burnouf T | Platelets; Microparticles; Extracellular Vesicles; COVID-19; SARS-Cov-2; VITT | Transfusion and Apheresis Science | 10.1016/j.transci.2022.103459 |
| 107 | Thromboplasminflammation in COVID-19 Coagulopathy: Three Viewpoints for Diagnostic and Therapeutic Strategies | Gando S; Wada T | COVID-19; Inflammation; Plasmin; SARS-Cov-2; Thrombin | Frontiers in Immunology | 10.3389/fimmu.2021.649122 |
| 108 | Carboxypeptidase B Blocks Ex Vivo Activation of the Anaphylatoxin-Neutrophil Extracellular Trap Axis in Neutrophils from COVID-19 Patients | Zhang Y; Han K; Du Cj; Li R; Liu Jy; Zeng H; Zhu Ll; Li A | COVID-19; Neutrophil Extracellular Trap; Anaphylatoxin; Carboxypeptidase B | Critical Care | 10.1186/s13054-021-03482-z |
| 109 | The Role of Direct Oral Anticoagulants in the Era of COVID-19: Are Antiviral Therapy and Pharmacogenetics Limiting Factors? | Roguljic H; Arambasic J; Nincevic V; Kuna L; Sesto I; Tabll A; Smolic R; Vcev A; Primorac-D; Wu Gy; Smolic M | NA | Croatian Medical Journal | 10.3325/cmj.2022.63.287 |
| 110 | Insights into Immunothrombotic Mechanisms in Acute Stroke Due to Vaccine-Induced Immune Thrombotic Thrombocytopenia | De Buhr N; Baumann T; Werlein C; Fingerhut L; Imker R; Meurer M; Goetz F; Bronzlik P; Kuehnel Mp; Jonigk Dd; Ernst J; Leotescu A; Gabriel Mm; Worthmann H; Lichtinghagen R; Tiede A; Von Koeckritz-Blickwede M; Falk Cs; Weissenborn K; Schuppner R; Grosse Gm | Immunothrombosis; Neutrophil Extracellular Traps (NETs); Stroke; Complement; Cytokines; Vaccination | Frontiers in Immunology | 10.3389/fimmu.2022.879157 |
| 111 | The Interplay Between Neutrophils, Complement, and Microthrombi in COVID-19 | Zuo Y; Kanthi Y; Knight Js; Kim Ahj | COVID-19; SARS-Cov-2; Neutrophil Extracellular Traps; NETs; Complement; Innate Immunity; Thrombotic Microangiopathy | Best Practice & Research in Clinical Rheumatology | 10.1016/j.berh.2021.101661 |
| 112 | Role of Neutrophils, Platelets, and Extracellular Vesicles and their Interactions in COVID-19-Associated Thrombopathy | Caillon A; Trimaille A; Favre J; Jesel L; Morel O; Kauffenstein G | COVID-19; Extracellular Vesicles; Neutrophils; Platelets; Therapeutic Strategy; Thrombosis | Journal of Thrombosis and Haemostasis | 10.1111/jth.15566 |
| 113 | COVID-19 and Thromboinflammation: Is there a role for Statins? | Ferrari F; Martins Vm; Teixeira M; Santos Rd; Stein R | COVID-19; Thrombosis; Inflammation; Atherosclerosis; Statins | Clinics | 10.6061/clinics/2021/e2518 |
| 114 | Upregulation of Pulmonary Tissue Factor, Loss of Thrombomodulin and Immunothrombosis in SARS-Cov-2 Infection | Francischetti Imb; Toomer K; Zhang Yf; Jani J; Siddiqui Z; Brotman Dj; Hooper Je; Kickler Ts | Thrombosis; Hemostasis; Coagulation; Ixolaris | Eclinicalmedicine | 10.1016/j.eclinm.2021.101069 |
| 115 | COVID-19 Hypothesis: Activated Protein C for Therapy of Virus-Induced Pathologic Thromboinflammation | Griffin Jh; Lyden P | Activated Protein C; Coronavirus; COVID-19; Cytokine; D-Dimer; SARS-Cov-2 | Research and Practice in Thrombosis and Haemostasis | 10.1002/rth2.12362 |
| 116 | A Review of Ischemic Stroke in COVID-19: Currently Known Pathophysiological Mechanisms | Tang Xh; Zheng F | COVID-19; Endothelial Dysfunction; Ischemic Stroke; SARS-Cov-2; Thrombosis | Neurological Sciences | 10.1007/s10072-021-05679-0 |
| 117 | Role of SARS-Cov-2-Induced Cytokines and Growth Factors in Coagulopathy and Thromboembolism | Ahmad F; Kannan M; Ansari Aww | COVID-19; Inflammation; Cytokines; Growth Factors; Platelet Activation; Coagulopathy; Thromboembolism | Cytokine & Growth Factor Reviews | 10.1016/j.cytogfr.2021.10.007 |
| 118 | Beyond Hemostasis: Platelet Innate Immune Interactions and Thromboinflammation | Mandel J; Casari M; Stepanyan M; Martyanov A; Deppermann C | Platelets; Hemostasis; Thrombosis; Neutrophils; Monocytes; Macrophages; Inflammation; NETs; COVID-19; Atherosclerosis; Cancer | International Journal of Molecular Sciences | 10.3390/ijms23073868 |
| 119 | Venous Thromboembolism in COVID-19 | Schulman S; Hu Y; Konstantinides S | COVID-19; D-Dimer; Risk Assessment Models; Venous Thromboembolism; Prophylaxis; Treatment | Thrombosis and Haemostasis | 10.1055/s-0040-1718532 |
| 120 | Transcriptional Reprogramming from Innate Immune Functions to a Pro-Thrombotic Signature by Monocytes in COVID-19 | Maher Ak; Burnham Kl; Jones Em; Tan Mmh; Saputil Rc; Baillon L; Selck C; Giang N; Arguello R; Pillay C; Thorley E; Short Ce; Quinlan R; Barclay Ws; Cooper N; Taylor Gp; Davenport Ee; Dominguez-Villar M | NA | Nature Communications | 10.1038/s41467-022-35638-y |
| 121 | Extrapulmonary Manifestations of COVID-19 | Gupta A; Madhavan Mv; Sehgal K; Nair N; Mahajan S; Sehrawat Ts; Bikdeli B; Ahluwalia N; Ausiello Jc; Wan Ey; Freedberg De; Kirtane Aj; Parikh Sa; Maurer Ms; Nordvig As; Accili D; Bathon Jm; Mohan S; Bauer Ka; Leon Mb; Krumholz Hm; Uriel N; Mehra Mr; Elkind Msv; Stone Gw; Schwartz A; Ho Dd; Bilezikian Jp; Landry Dw | NA | Nature Medicine | 10.1038/s41591-020-0968-3 |
| 122 | TIE2 Activation Protects Against Prothrombotic Endothelial Dysfunction in COVID-19 | Schmaier Aa; Hurtado Gmp; Manickas-Hill Zj; Sack Kd; Chen Sy; Bhambhani V; Quadir J; Nath Ak; Collier Ary; Ngo D; Barouch Dh; Shapiro Ni; Gerszten Re; Yu X; Peters Kg; Flaumenhaft R; Parikh Sm | COVID-19; Coagulation; Vascular Biology | JCI Insight | 10.1172/jci.insight.151527 |
| 123 | A Tricompartmental Model of Lung Oxygenation Disruption to Explain Pulmonary and Systemic Pathology in Severe COVID-19 | Mcgonagle D; Bridgewood C; Meaney Jfm | NA | Lancet Respiratory Medicine | 10.1016/S2213-2600(21)00213-7 |
| 124 | Viral-Induced Inflammatory Coagulation Disorders: Preparing for Another Epidemic | Iba T; Levy Jh; Levi M | Viral Hemorrhagic Fever; COVID-19; Anticoagulation; Disseminated Intravascular Coagulation; Endothelial Cells | Thrombosis and Haemostasis | 10.1055/a-1562-7599 |
| 125 | Platelet Activation and Thrombosis in COVID-19 | Iba T; Wada H; Levy Jh | COVID-19; Platelet; Thrombosis; Von Willebrand Factor; Platelet Factor 4; P-Selectin; C-Type Lectin-Like Receptor 2; Antiplatelet | Seminars in Thrombosis and Hemostasis | 10.1055/s-0042-1749441 |
| 126 | COVID-19 and Pulmonary Angiogenesis: The Possible Role of Hypoxia and Hyperinflammation in the Overexpression of Proteins Involved in Alveolar Vascular Dysfunction | Miggiolaro Afrs; Da Silva Fpg; Wiedmer Db; Godoy Tm; Borges Nh; Piper Gw; Oricil Agg; Klein Ck; Hlatchuk Ec; Dagostini Jch; Collete M; Arantes Mp; D'Amico Rc; Dutra Aa; De Azevedo Mlv; De Noronha L | SARS-Cov-2; Endothelial Dysfunction; Angiogenesis; Microthrombi; Immunohistochemistry | Viruses-Basel | 10.3390/v15030706 |
| 127 | The Vascular Endothelium: The Cornerstone of Organ Dysfunction in Severe SARS-Cov-2 Infection | Pons S; Fodil S; Azoulay E; Zafrani L | SARS-Cov-2; COVID-19; Endothelial Cells; Endothelial Dysfunction; Cytokines; Thrombosis | Critical Care | 10.1186/s13054-020-03062-7 |
| 128 | Molecular mechanisms of vasculopathy and coagulopathy in COVID-19 | Al-Gburi S; Beissert S; Gunther C | Angiotensin-II-Converting-Enzyme-2 (ACE2); Coagulopathy; Endothelitis; SARS-Cov-2; Sex Differences; Vasculopathy | Biological Chemistry | 10.1515/hsz-2021-0245 |
| 129 | Mannose-Binding Lectin is Associated with Thrombosis and Coagulopathy in Critically Ill COVID-19 Patients | Eriksson O; Hultstrom M; Persson B; Lipcsey M; Ekdahl Kn; Nilsson B; Frithiof R | Thrombosis; COVID-19; Complement System; Mannose-Binding Lectin | Thrombosis and Haemostasis | 10.1055/s-0040-1715835 |
| 130 | Pharmacological Agents Targeting Thromboinflammation in COVID-19: Review and Implications for Future Research | Bikdeli B; Madhavan Mv; Gupta A; Jimenez D; Burton Jr; Nigoghossian Cd; Chuich T; Nouri Sn; Dreyfus I; Driggin E; Sethi S; Sehgal K; Chatterjee S; Ageno W; Madjid M; Guo Yt; Tang Lv; Hu Y; Bertoletti L; Giri J; Cushman M; Quere I; Dimakakos Ep; Gibson Cm; Lippi G; Favaloro Ej; Fareed J; Tafur Aj; Francese Dp; Batra J; Falanga A; Clerkin Kj; Uriel N; Kirtane A; Mclintock C; Hunt Bj; Spyropoulos Ac; Barnes Gd; Eikelboom Jw; Weinberg I; Schulman S; Carrier M;Piazza G;Beckman Ja;Leon Mb;Stone Gw;Rosenkranz S;Goldhaber Sz;Parikh Sa;Monreal M;Krumholz Hm;Konstantinides Sv;Weitz Ji;Lip Gyh | Coronavirus Disease 2019; Thrombosis; Inflammation; Fibrinolytic Therapy; Anticoagulation; Immunomodulator; Antithrombin; Thrombomodulin | Thrombosis and Haemostasis | 10.1055/s-0040-1713152 |
| 131 | Platelet Reactivity and Inflammatory Phenotype Induced by Full-Length Spike SARS-CoV-2 Protein and Its RBD Domain | Cano-Mendez A; Garcia-Larragoiti N; Damian-Vazquez M; Guzman-Cancino P; Lopez-Castaneda S; Ochoa-Zarzosa A; Viveros-Sandoval Me | Platelet Activation; Platelet Aggregation; SARS-Cov-2; Spike Protein; RBD Domain | International Journal of Molecular Sciences | 10.3390/ijms232315191 |
| 132 | A Vicious Cycle: In Severe and Critically Ill COVID-19 Patients | Huang Pf; Zuo Qw; Li Y; Oduro Pk; Tan Fx; Wang Yy; Liu Xh; Li J; Wang Ql; Guo F; Yang L | COVID-19; PAI-1; IL-6; Inflammatory Reaction; Venous Thrombosis; Tocilizumab; Endothelial Cells | Frontiers in Immunology | 10.3389/fimmu.2022.930673 |
| 133 | Endothelium Infection and Dysregulation by SARS-CoV-2: Evidence and Caveats in COVID-19 | Bernard I; Limonta D; Mahal Lk; Hobman Tc | COVID-19; SARS-Cov-2; ACE2; RAAS; Bradykinin-Kallikrein Pathway; ADAM17; Endothelial Dysfunction; Pericyte; Immunothrombosis; Therapeutics | Viruses-Basel | 10.3390/v13010029 |
| 134 | The significance of surface neutrophilic MPO expression level in NETosis and NETosis-associated coagulopathies in covid-19 infected patients | Jamali E; Abbasi M; Tayer Ah; Monfared Aa; Tandel P; Tamaddon G; Kazerooni Es; Rakhshandehroo S; Ranjbaran R | Surface Myeloperoxidase; NETosis; Coagulopathies | Blood Cells Molecules and Diseases | 10.1016/j.bcmd.2022.102676 |
| 135 | Molecular Basis for Paradoxical Activities of Polymorphonuclear Neutrophils in Inflammation/Anti-Inflammation, Bactericide/Autoimmunity, Pro-Cancer/Anticancer, and Antiviral Infection/SARS-Cov-II-Induced Immunothrombotic Dysregulation | Wu Th; Hsieh Sc; Li Th; Lu Ch; Liao Ht; Shen Cy; Li Kj; Wu Ch; Kuo Ym; Tsai Cy; Yu Cl | Polymorphonuclear Neutrophil; Mitogen-Induced Cell-Mediated Cytotoxicity; Antibody-Dependent Cell-Mediated Cytotoxicity; Neutrophil Extracellular Traps; Ectosomes; Exosomes; Trogocytosis; SARS-Cov-2 Pandemic; Immune Homeostasis; Immunothrombosis | Biomedicines | 10.3390/biomedicines10040773 |
| 136 | An increase in CD62L^dim^ neutrophils precedes the development of pulmonary embolisms in COVID-19 patients | Spijkerman R; Jorritsma Nkn; Bongers Sh; Bindels Bjj; Jukema Bn; Hesselink L; Hietbrink F; Leenen Lph; Van Goor Hmr; Vrisekoop N; Kaasjager Kah; Koenderman L | CD62L; COVID-19; Intensive Care Unit; L-Selectin; Neutrophils; SARS-Cov-2; Thrombosis; Pulmonary Embolism | Scandinavian Journal of Immunology | 10.1111/sji.13023 |
| 137 | Increased prevalence of deep vein thrombosis and mortality in patients with COVID-19 at a referral center in Brazil | De Godoy Jmp; Russeff Gjd; Cunha Ch; Sato Dy; Silva Dfd; De Godoy Hjp; Da Silva Mom; Amorim H; Soares Mml; Godoy Mdg | Prevalence; Deep Vein; Thrombosis; Mortality; COVID-19 | Phlebology | 10.1177/02683555211041931 |
| 138 | Prognostic Value of Thrombin Generation Parameters in Hospitalized COVID-19 Patients | De La Morena-Barrio Me; Bravo-Perez C;Minano A; De La Morena-Barrio B; Fernandez-Perez Mp; Bernal E; Gomez-Verdu Jm; Herranz Mt; Vicente V; Corral J; Lozano Ml | NA | Scientific Reports | 10.1038/s41598-021-85906-y |
| 139 | COVID-19 in the Healthy Patient Population: Demographic and Clinical Phenotypic Characterization and Predictors of In-Hospital Outcomes | Botero Dmr; Omar Ams; Sun Hk; Mantri N; Fortuzi K; Choi Y; Adrish M; Nicu M; Bella Jn; Chilimuri S | Follow-Up Studies; Inflammation; Middle Aged; Mortality; Thromboembolism | Arteriosclerosis Thrombosis and Vascular Biology | 10.1161/ATVBAHA.120.314845 |
| 140 | Role of Vitamin D in Treating COVID-19-Associated Coagulopathy: Problems and Perspectives | Sengupta T; Majumder R; Majumder S | Vitamin D; COVID-19; Coagulation; Inflammation; Hypercoagulability; Thromboinflammation; Thrombosis; Venous Thromboembolism (VTE) | Molecular and Cellular Biochemistry | 10.1007/s11010-021-04093-6 |
| 141 | Clinical and Computed Tomography Characteristics of COVID-19 Associated Acute Pulmonary Embolism: A Different Phenotype of Thrombotic Disease? | Van Dam Lf; Kroft Ljm; Van Der Wal Li; Cannegieter Sc; Eikenboom J; De Jonge E; Huisman Mv; Klok Fa | Pulmonary Embolism; COVID-19; Computed Tomography; Thrombosis | Thrombosis Research | 10.1016/j.thromres.2020.06.010 |
| 142 | Hematological Abnormalities in Patients with the SARS-Cov-2 (COVID-19) and its Prognostic Implications | Castellanos-Sinco Hb; Pellon-Tellez K; Rodriguez-Fuentes K; Mendoza-Oliva M; Baltodano-Martinez Y; Munoz-Vega J; Tapia-Bravo M; Telleria-Arispe C; De La Mora-Estrada M; Ramos-Penafiel C; Zazueta-Pozos F; Barranco-Lampon G; Montano-Figueroa E; Martinez-Murillo C | Hematology; Coagulation; D-Dimers; Thrombocytopenia | Gaceta Medica De Mexico | 10.24875/GMM.M21000465 |
| 143 | Deciphering The Role of Monocyte and Monocyte Distribution Width (MDW) in COVID-19: An Updated Systematic Review and Meta-Analysis | Ligi D; Lo Sasso B; Henry Bm; Ciaccio M; Lippi G; Plebani M; Mannello F | COVID-19; Histone; Meta-Analysis; Monocyte; Monocyte Distribution Width; NETosis; SARS-Cov-2; Systematic Review; Thrombo-Inflammation | Clinical Chemistry and Laboratory Medicine | 10.1515/cclm-2022-0936 |
| 144 | Platelet Activation State in Early Stages of COVID-19 | Consolo F; Della Valle P; Saracino M; Bonora M; Donadoni G; Ciceri F; Tresoldi M; D'Angelo A; Landoni G; Zangrillo A | Platelet Activation; COVID-19; Thromboinflammation | Minerva Anestesiologica | 10.23736/S0375-9393.22.16054-2 |
| 145 | Immune Cartography of Macrophage Activation Syndrome in the COVID-19 Era | Mcgonagle D; Ramanan Av; Bridgewood C | NA | Nature Reviews Rheumatology | 10.1038/s41584-020-00571-1 |
| 146 | Coagulopathy of Dengue and COVID-19: Clinical Considerations | Islam A; Cockcroft C; Elshazly S; Ahmed J; Joyce K; Mahfuz H; Islam T; Rashid H; Laher I | COVID-19; Cross-Reactivity; Dengue; Haemorrhage; Thrombocytopenia; Thrombosis | Tropical Medicine and Infectious Disease | 10.3390/tropicalmed7090210 |
| 147 | Platelet-Monocyte Interaction Amplifies Thromboinflammation Through Tissue Factor Signaling in COVID-19 | Hottz Ed; Martins-Goncalves R; Palhinha L; Azevedo-Quintanilha Ig; De Campos Mm; Sacramento Cq; Temerozo Jr; Soares Vc; Dias Ssg; Teixeira L; Castro I; Righy C; Souza Tml; Kurtz P; Andrade Bb; Nakaya Hi; Monteiro Rq; Bozza Fa; Bozza Pt | NA | Blood Advances | 10.1182/bloodadvances.2021006680 |
| 148 | Platelet Activation and Partial Desensitization are Associated with Viral Xenophagy in Patients with Severe COVID-19 | Garcia C; Duong Ja; Poeette M; Ribes A; Payre B; Memier V; Sie P; Minville V; Voisin S; Payrastre B; Vardon-Bounes F | NA | Blood Advances | 10.1182/bloodadvances.2022007143 |
| 149 | Increased Lung Immune Metabolic Activity in COVID-19 Survivors | Rodrigues Rs; Ribeiro Gm; Barreto Mm; Zin Wa; De Toledo-Mendes J; Martins Pag; De Almeida Sa; Basilio R; Martins-Goncalves R; Hottz Ed; Bozza Pt; Bozza Fa; Carvalho Ars; Rosado-De-Castro Ph | COVID-19 Pneumonia; Endothelial Cell Activation; Immunometabolism; Long COVID; PET/CT; Pulmonary Inflammation | Clinical Nuclear Medicine | 10.1097/RLU.0000000000004376 |
| 150 | Thromboembolic Events in Deceased Patients with Proven SARS-Cov-2 Infection: Frequency, Characteristics and Risk Factors | Voigtlaender M; Edler C; Gerling M; Schadler J; Ondruschka B; Schroder As; Sperhake J; Ehrhardt S; Wang L; Haddad M; Kiencke V; Renne T; Roedl K; Kluge S; Wichmann D; Langer F | COVID-19; Thromboembolism; Autopsy; Hereditary Thrombophilia; Body Mass Index; Intensive Care Unit | Thrombosis Research | 10.1016/j.thromres.2022.08.021 |
| 151 | Potential Mechanisms of Vaccine-Induced Thrombosis | Marietta M; Coluccio V; Luppi M | Ad26.COV2.S; Autoimmune Heparin-Induced Thrombocytopenia; BNT162b; COVID-19 vaccines; ChAdOx1 nCoV-19; Vaccine- Induced Immune Thrombocytopenia and Thrombosis. | European Journal of Internal Medicine | 10.1016/j.ejim.2022.08.002 |
| 152 | Prothrombotic Phenotype in COVID-19: Focus on Platelets | Barale C; Melchionda E; Morotti A; Russo I | Platelet Activation; COVID-19; SARS-Cov-2; Thrombosis; Inflammation; Immunothrombosis | International Journal of Molecular Sciences | 10.3390/ijms222413638 |
| 153 | COVID-19 Microthrombosis: Unusually Large VWF Multimers are a Platform for Activation of the Alternative Complement Pathway Under Cytokine Storm | Fujimura Y; Holland Lz | COVID-19; VWF; ADAMTS13; Complement Activation; Endotheliopathy; Microthrombosis | International Journal of Hematology | 10.1007/s12185-022-03324-w |
| 154 | Pathogeny of Cerebral Venous Thrombosis in SARS-Cov-2 Infection Case Reports | Guendouz C; Quenardelle V; Riou-Comte N; Welfringer P; Wolff V; Zuily S; Jager L; Selton Lh; Mione G; Pop R; Gory B; Richard S | Cerebral Infarction; Cerebral Venous Thrombosis; COVID-19; SARS-Cov-2; Stroke | Medicine | 10.1097/MD.0000000000024708 |
| 155 | COVID-19-Associated Pulmonary Embolism: Review of the Pathophysiology, Epidemiology, Prevention, Diagnosis, and Treatment | Ortega-Paz L; Talasaz Ah; Sadeghipour P; Potpara Ts; Aronow Hd; Jara-Palomares L; Sholzberg M; Angiolillo Dj; Lip Gyh; Bikdeli B | SARS-Cov-2; Coronavirus Disease 2019; Long COVID; Pulmonary Embolism; Venous Thromboembolism; Anticoagulant Therapy | Seminars in Thrombosis and Hemostasis | 10.1055/s-0042-1757634 |
| 156 | Thrombocytopathy and Endotheliopathy: Crucial Contributors to COVID-19 Thromboinflammation | Gu Sx; Tyagi T; Jain K; Gu Vw; Lee Sh; Hwa Jm; Kwan Jm; Krause Ds; Lee Ai; Halene S; Martin Ka; Chun Hj; Hwa J | NA | Nature Reviews Cardiology | 10.1038/s41569-020-00469-1 |
| 157 | COVID-19 Associated Thromboinflammation of Renal Capillary: Potential Mechanisms and Treatment | Chen Xj; Yu Cy; Jing Hj; Wang Cx; Zhao Xy; Zhang Jm; Zhang Sq; Liu H; Xie Rj; Shi Jl | COVID-19; Inflammation; Renal Capillary Thrombosis; Antithrombotic Therapy; Graded Management | American Journal of Translational Research | NA (Am J Transl Res 2020;12(12):7640-7656) |
| 158 | Pathology of Lung-Specific Thrombosis and Inflammation in COVID-19 | Khismatullin Rr; Ponomareva Aa; Nagaswami C; Ivaeva Ra; Montone Kt; Weisel Jw; Litvinov Ri | Blood Coagulation; COVID-19; Inflammation; Lungs; Thrombosis | Journal of Thrombosis and Haemostasis | 10.1111/jth.15532 |
| 159 | Complement Overactivation and Consumption Predicts In-Hospital Mortality in SARS-Cov-2 Infection | Sinkovits G; Mezo B; Reti M; Muller V; Ivanyi Z; Gal J; Gopcsa L; Remenyi P; Szathmary B; Lakatos B; Szlavik J; Bobek I; Prohaszka Zz; Forhecz Z; Csuka D; Hurler L; Kajdacsi E; Cervenak L; Kiszel P; Masszi T; Valyi-Nagy I; Prohaszka Z | SARS-Cov-2 Infection; Mortality; Severity; Complement System; Coronavirus Disease (COVID-19); Complement Activation and Consumption | Frontiers in Immunology | 10.3389/fimmu.2021.663187 |
| 160 | Platelets Promote Thromboinflammation in SARS-Cov-2 Pneumonia | Taus F; Salvagno G; Cane S; Fava C; Mazzaferri F; Carrara E; Petrova V; Barouni Rm; Dima F; Dalbeni A; Romano S; Poli G; Benati M; De Nitto S; Mansueto G; Iezzi M; Tacconelli E; Lippi G; Bronte V; Minuz P | Blood Platelets; Inflammation; Interferons; Monocytes; Thrombosis | Arteriosclerosis Thrombosis and Vascular Biology | 10.1161/ATVBAHA.120.315175 |
| 161 | Pathophysiological Mechanisms of Thrombosis in Acute and Long COVID-19 | Jing Hj; Wu Xm; Xiang Mq; Liu Lj; Novakovic Va; Shi Jl | Inflammation; Immunothrombosis; Anti-Inflammatory Treatment; Antithrombotic Therapy; Long COVID-19 | Frontiers in Immunology | 10.3389/fimmu.2022.992384 |
| 162 | Disease Severity in Moderate-To-Severe COVID-19 is Associated with Platelet Hyperreactivity and Innate Immune Activation | Jakobs K; Reinshagen L; Puccini M; Friebel J; Wilde Acb; Alsheik A; Rroku A; Landmesser U; Haghikia A; Krankel N; Rauch-Krohnert U | COVID-19; Platelet Hyperactivity; Immunothrombosis; Inflammation; Platelet-Leucocyte Aggregates; Disease Severity; Survival | Frontiers in Immunology | 10.3389/fimmu.2022.844701 |
| 163 | Gegen Qinlian pills alleviate carrageenan-induced thrombosis in mice model by regulating the HMGB1/NF-κB/NLRP3 signaling | Wei Xh; Zhang Bp; Wei Fy; Ding Mz; Luo Zy; Han Xl; Tan Xm | COVID-19; Gegen Qinlian Pills; HMGB1/NF-κB/NLRP3 Signaling; Hyperinflammatory; Thrombosis | Phytomedicine | 10.1016/j.phymed.2022.154083 |
| 164 | Impaired Coagulation, Liver Dysfunction and COVID-19: Discovering an Intriguing Relationship | D'Ardes D; Boccatonda A; Cocco G; Fabiani S; Rossi I; Bucci M; Guagnano Mt; Schiavone C; Cipollone F | COVID-19; SARS-Cov-2; Liver; Coagulation | World Journal of Gastroenterology | 10.3748/wjg.v28.i11.1102 |
| 165 | Thrombotic Complications in Severe COVID-19: Focus on Venous Thromboembolism, Thromboprophylaxis and Anticoagulation | Porres-Aguilar M; Guerrero-De Leon Mc; Grimaldo-Gomez Fa; Izaguirre-Avila R; Cabrera-Rayo A; Santos-Martinez Le; Sanchez-Medina Jr; Porres-Munoz M; Carrillo-Esper R | Anticoagulation; COVID-19; Venous Thromboembolism; Thromboprophylaxis; Thrombosis | Cirugia Y Cirujanos | 10.24875/CIRU.20000879 |
| 166 | The Aetiopathogenesis of Vaccine-Induced Immune Thrombotic Thrombocytopenia | Toh Ch; Wang Gz; Parker Al | COVID-19; Fc-gamma-RIIa; Adenovirus Vaccines; Platelet Factor-4; Vaccine-Induced Immune Thrombotic Thrombocytopenia | Clinical Medicine | 10.7861/clinmed.2022-0006 |
| 167 | Endothelial, Immunothrombotic, and Inflammatory Biomarkers in the Risk of Mortality in Critically Ill COVID-19 Patients: The Role of Dexamethasone | Keskinidou C; Vassiliou Ag; Zacharis A; Jahaj E; Gallos P; Dimopoulou I; Orfanos Se; Kotanidou A | COVID-19; ICU; Dexamethasone; Mortality; Endothelial Dysfunction; Coagulation; Inflammation; suPAR; Presepsin; sVCAM-1 | Diagnostics | 10.3390/diagnostics11071249 |
| 168 | Neutrophil Extracellular Traps Infiltrate the Lung Airway, Interstitial, and Vascular Compartments in Severe COVID-19 | Radermecker C; Detrembleur N; Guiot J; Cavalier E; Henket M; D'Emal C; Vanwinge C; Cataldo D; Oury C; Delvenne P; Marichal T | COVID-19, Infectious Disease and Host Defense, Innate Immunity and Inflammation, Mucosal Immunology | Journal of Experimental Medicine | 10.1084/jem.20201012 |
| 169 | Circulating Markers of Neutrophil Extracellular Traps Are of Prognostic Value in Patients with COVID-19 | Ng H;Havervall S;Rosell A;Aguilera K;Parv K;Von Meijenfeldt Fa;Lisman T;Mackman N;Thalin C;Phillipson M | Cytokines; Extracellular Traps; Fibrinolysis; Histone; Immunothrombosis; SARS-Cov2; Thrombosis | Arteriosclerosis Thrombosis and Vascular Biology | 10.1161/ATVBAHA.120.315267 |
| 170 | The Roles of Platelets in COVID-19-Associated Coagulopathy and Vaccine-induced Immune Thrombotic Thrombocytopenia | Iba T; Levy Jh | COVID-19; Platelet; Coagulopathy; Thrombosis; Thrombocytopenia | Trends in Cardiovascular Medicine | 10.1016/j.tcm.2021.08.012 |
| 171 | COVID-19. Immunothrombosis and The Gastrointestinal Tract | Cienfuegos Ja; Almeida A; Sanchez-Justicia C | COVID-19; Colon Ischemia; Intravascular Disseminated Coagulation; Immunothrombosis | Revista Espanola De Enfermedades Digestivas | 10.17235/reed.2020.7292/2020 |
| 172 | Selective Inhibition of Thromboinflammation in COVID-19 By BTK inhibitors | Siess W; Von Hundelshausen P; Lorenz R | BTK; COVID-19; Fc Gamma-Receptor; Neutrophils; Platelets; Thromboinflammation | Platelets | 10.1080/09537104.2020.1809647 |
| 173 | Change in Platelet Indices in Patients with Coronavirus Disease-2019 (COVID-19): A Reflection of Platelet Activation and Contribution to Immunothrombosis? | Mezgebe M; Jacobson Bf; Mayne Es; Louw S | COVID-19; Immunothrombosis; Laboratory Parameters; Platelets | International Journal of Laboratory Hematology | 10.1111/ijlh.13705 |
| 174 | A Rationale for Blocking Thromboinflammation in COVID-19 With BTK inhibitors | Nicolson Plr; Welsh Jd; Chauhan A; Thomas Mr; Kahn Ml; Watson Sp | BTK; COVID-19; Platelets; Thromboinflammation | Platelets | 10.1080/09537104.2020.1775189 |
| 175 | Microvascular COVID-19 Lung Vessels Obstructive Thromboinflammatory Syndrome (MicroCLOTS): A New Variant of Thrombotic Microangiopathy? | Bobrova L; Kozlovskaya N; Korotchaeva Y; Bobkova I; Kamyshova E; Moiseev S | NA | Critical Care and Resuscitation | 10.1016/S1441-2772(23)00399-X |
| 176 | The COVID-19 Arterial Thromboembolic Complications: From Inflammation to Immunothrombosis Through Antiphospholipid Autoantibodies | Roncati L; Manenti A; Manco G; Farinetti A; Mattioli A | NA | Annals of Vascular Surgery | 10.1016/j.avsg.2020.12.006 |
| 177 | Thromboinflammatory State and Venous Thromboembolic Events in Patients with Coronavirus Disease 2019 Admitted to a Nonintensive Care Unit: A Prospective Study | Fortini A; Beltrame C; Faraone A; Iandelli S; Zaccagnini G; Lo Forte A | NA | Polish Archives of Internal Medicine-Polskie Archiwum Medycyny Wewnetrznej | 10.20452/pamw.15625 |
| 178 | COVID-19: Angiotensin II in Development of Lung Immunothrombosis and Vasculitis Mimics | Lloyd-Jones G; Oudkerk M | NA | Lancet Rheumatology | 10.1016/S2665-9913(21)00068-0 |
| 179 | Value of Hypocalcemia and Thromboinflammatory Biomarkers for Prediction of COVID-19 Severity During the Second Wave: Were All the Waves the Same? | De Guadiana-Romualdo Lg; Ramos-Arenas V; Mulero Mdr; Olivo Mh; Campos-Rodriguez V; Martinez Mg; Braquehais Msr; Consuegra-Sanchez L; Morales Mg; Albaladejo-Oton Md | Ionized Calcium; Prognosis; Second Wave; Severity COVID-19 | Clinical Chemistry and Laboratory Medicine | 10.1515/cclm-2021-0996 |
| 180 | Severe COVID-19 as a Virus-Independent Immunothrombotic Process | Cherian R; Tung Ml; Chandra B | NA | Lancet Rheumatology | 10.1016/S2665-9913(22)00033-9 |
